# Research landscape and trends of melanoma immunotherapy: A bibliometric analysis

**DOI:** 10.3389/fonc.2022.1024179

**Published:** 2023-01-09

**Authors:** Yanhao Liu, Lan Yu, Yanjiao Liang, Xi Cheng, Shu Jiang, Haiming Yu, Zhen Zhang, Linlin Lu, Baozhen Qu, Yuxian Chen, Xiaotao Zhang

**Affiliations:** Department of Radiation Oncology, The Affiliated Qingdao Central Hospital of Qingdao University, Qingdao, China

**Keywords:** bibliometric analysis, melanoma, PD1/PDL1, clinical trials, CTLA-4, adoptive cell therapy, immunotherapy, tumor mutation burden

## Abstract

**Background:**

Immunotherapy for lung cancer has been a hot research area for years. This bibliometric analysis was intended to present research trends on melanoma immunotherapy.

**Method:**

On April 1, 2022, the authors identified 2,109 papers on melanoma immunotherapy using the Web of Science and extracted their general information and the total number of citations. The authors then conducted a bibliometric analysis to present the research landscape, clarify the research trends, and determine the most cited papers (top-papers) as well as major journals on melanoma immunotherapy. Subsequently, recent research hotspots were identified by analyzing the latest articles in major journals.

**Results:**

The total and median number of citations of these 2,109 papers on melanoma immunotherapy was 137,686 and 11, respectively. “Improved survival with ipilimumab in patients with metastatic melanoma” by Hodi et al. was the most cited paper (9,824 citations). Among the journals, the top-paper number (16), average citations per paper (2,510.7), and top-papers rate (100%) of *New England Journal of Medicine* were the highest. Corresponding authors represented the USA took part in most articles (784). Since 2016, the hottest research area has changed from CTLA-4 to PD-1.

**Conclusions:**

This bibliometric analysis comprehensively and quantitatively presents the research trends and hotspots based on 2,109 relevant publications, and further suggests future research directions. The researchers can benefit in selecting journals and in finding potential collaborators. This study can help researchers gain a comprehensive impression of the research landscape, historical development, and current hotspots in melanoma immunotherapy and can provide inspiration for future research.

## 1 Introduction

Melanoma, as the most invasive and deadly cutaneous malignant tumor, was mainly occurs in the skin that exposed to ultraviolet injury (1). A history of ultraviolet exposure of melanoma commonly induces a high tumor mutational burden (TMB), which lead to a high neoantigen load (2). Furthermore, a high neoantigen load may potentially facilitate the immune system to recognize the tumor (3). Thus, high TMB is usually related with a potent anti-tumor response following immunotherapy (3). Therefore, among the treatment modalities of melanoma, immunotherapy has always been an area of interest.

Before 2010, massive-dose interleukin-2 (IL-2) therapy was the most commonly used immunotherapy for melanoma. Some earlier trials reported the 5-year overall survival (OS) rates of patients with metastatic melanoma of 8-23% (4–6). A large retrospective study demonstrated the OS at 5-year of metastatic melanoma was less than 10% at that time (7). Encouragingly, the outcome of melanoma patients has dramatically improved in recent years by the development of agents for immune checkpoint blockade (ICB) (that target programmed cell death 1 [PD-1] and cytotoxic T lymphocyte-associated protein 4 [CTLA-4] coinhibitory receptors) and BRAF/(mitogen-activated protein kinase) MAPK kinase (MEK) targeted therapy (8). The 5-year OS rate of patients with metastatic melanoma who received single-agent PD-1 blockade or MEK targeted therapy has risen to 34%–44% (9–11). Furthermore, the patients received nivolumab-plus-ipilimumab therapy have achieved a 5-year OS rate of as high as 52% (11).

The new modalities, and especially ICB, have radically changed the management strategies for advanced melanoma. Given its improved rate, depth, and durability of response, immunotherapy has been the preferred 1st-line therapy for a large portion of patients with advanced melanoma (8). However, the overall response rates (ORR) are still dissatisfactory, and the treatment choices in patients who are resistant to immunotherapy and targeted therapy are limited. Further research is needed to overcome primary and acquired resistance, establish new approaches, and develop robust predictive biomarkers. Immunotherapy for melanoma has been a growing area of research since 2010, with thousands of studies published. It is therefore a challenge to recognize the most influential studies and systematically comprehend the research landscape and trends. At present, the research on immunotherapy for advanced melanoma has achieved remarkable results and further advances are under way. A comprehensive and quantified analysis is therefore needed to summarize and collate the knowledge, demonstrate the current research hotspots, and indicate future research trends.

Classic reviews show the progress in a specific research field and are influenced by the authors’ own knowledge and opinions. Oppositely, bibliometric analyses, as a quantitative and objective analytical method based on thousands of papers, can comprehensively present the research status of an entire area (12, 13). In addition, bibliometric analyses based on multiple software, such as VOSviewer and CiteSpace, can visualize the information and knowledge to intuitively present the features of a research area (14–16). Furthermore, the recognition of core journals, publications, and authors by the co-citation and collaboration network can aid researchers to understand the historical development and latest research panorama. In recent years, several bibliometric analyses related to melanoma have been published. However, only three have been published in the past 5 years; among them, two only focused on uveal melanoma (17, 18). The other study intended to determine the trends in research and public interest, but only analyzed 15 top-cited papers and presented Google Trends (19). Therefore, bibliometric analysis on melanoma immunotherapy is lacking, and a rigorous, in-depth, and useful bibliometric analysis is warranted.

The current bibliometric analysis addressed the following research questions on melanoma immunotherapy: 1) What are the historical research trends, current research status, and future research directions? 2) Which are the most influential publications and major journals? 3) Which are the top contributing countries, institutions, and authors? This study focused on original research pertaining to melanoma immunotherapy and was intended to help researchers get a comprehensive picture of the research panorama, historical development, and recent hotspots in this area. The authors analyzed papers published since 2010 and identified the most influential studies to demonstrate the important advances and research focus. In addition, a further analysis based on the newest papers in major journals was carried out to present the latest research trends and predict future research direction.

The rest of the paper is organized as follows: Section 2 describes the methods used for literature search, statistics, and diagramming. Section 3 presents the results of the bibliometric analysis on publications, journals, countries/regions, institutions, authors, and research trends. Section 4 discusses advances in melanoma immunotherapy, the results of the analysis, and limitations, followed by the conclusions in Section 5.

## 2 Methods

### 2.1 Paper selection

The authors selected the Science Citation Indexing Expanded database of the Web of Science to search for publications on melanoma immunotherapy. This database is commonly used and suitable for bibliometric analyses; it contains more than ten thousand impactful journals and provides exhaustive citation data (20). Moreover, the accuracy of document type labeling of the Web of Science has been demonstrated to be superior to that of other databases such as Scopus (21). On April 1, 2022, the authors carried out a literature retrieval. The document type was restricted to original research and the publication year was restricted to 2010-2022. The search strategy (**Supplementary Material S1**) was rigorously defined by including various synonyms of the keywords and excluding reviews and meta-analyses. The authors conducted multiple tests and corrections to improve the precision of the search strategy. In this study, immunotherapy for melanoma included ICB, adoptive cell therapy (ACT), talimogene laherparepvec (T-VEC), therapeutic vaccine, and IL-2. Because “IL-2” was outdated, it was not included in the search string. The search results were reviewed to eliminate irrelevant papers. Duplicated publications were excluded by comparing the digital object unique identifiers and PubMed unique identifiers of the publications. Subsequently, the papers were ranked by the number of citations to demonstrate the top-100 most cited papers (top-papers) on melanoma immunotherapy. The authors then extracted the following data: title, abstract, keywords, author, country/region, institution; journal, publication time, and total citation number. According to citation number and publication time, the authors calculated the average citation number per year of each paper. Given hundreds of papers was newly published, the authors defined the average citations per year as the citations per month × 12.

In order to analyze the research trends on melanoma immunotherapy, the authors classified the paper titles by specific treatments. In addition, the authors searched papers related to IL-2 for melanoma to compare it with other treatments. The paper numbers and total citations per year were calculated and visualized for each treatment.

The authors then identified the journals which published top-papers and calculated the top-cited paper rates (TPR) of the journals (percentage of top-papers among all papers in a journal). The time span extended from 2010 to 2020 (the year of publication of the most recent top-cited paper). Journals with a TPR >5% were considered as major journals on melanoma immunotherapy. The original studies published in the major journals since 2020 were analyzed to present the latest research trends and predict future research direction.

### 2.2 Statistical analysis

The authors used Excel 2019 (Microsoft, WA, USA) for basic statistics and table preparation. The “bibliometrix” package of R (v4.1.1) was applied for bibliometric map plotting and analyzing. The VOSviewer (v1.6.17) was applied to visualization the citation, collaboration, or co-occurrence relationship between the journals, references, countries, institutions, authors, and keywords. A website (https://bibliometric.com) was used to visualize the cooperation between countries and another website (https://www.citexs.com) was used to visualize the trends of keyword frequencies. CiteSpace software (v6.1.R1) was applied to analyze and visualize outbreak references and keywords to present the research trends.

## 3 Results

**Figure 1:**
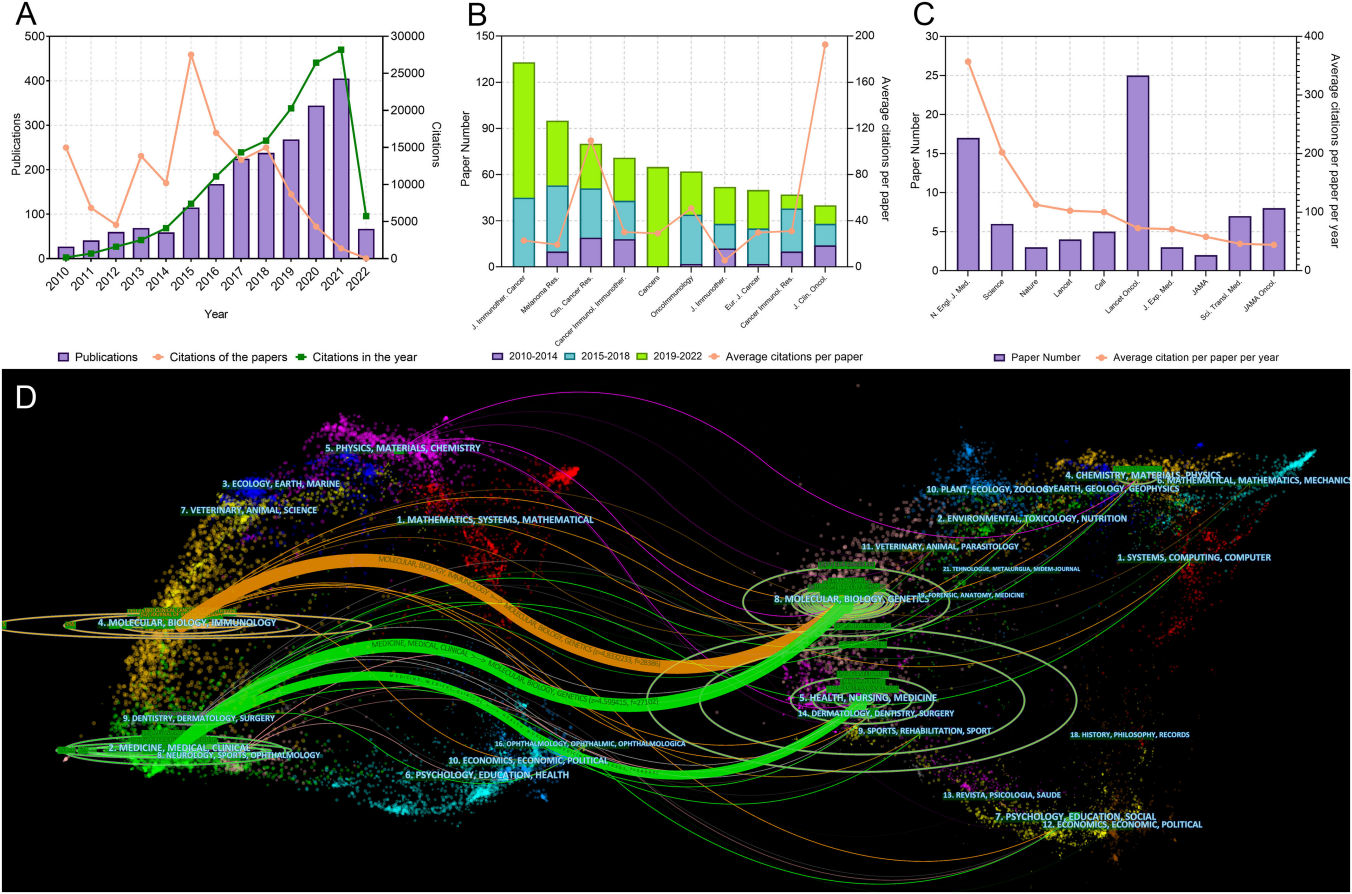
**(A)** Publication and citation number from 2010 to 2022 of the papers on melanoma immunotherapy. The green line indicates the total citations of papers published each year. The orange line indicates the total citations of all papers each year. **(B)** Paper numbers and average citations per paper of the top-10 productive journals. **(C)** Top-10 journals with the most citations per paper per year. **(D)** The dual-map overlay of journal categories. The left nodes represent citing journals and the right nodes represent cited journals. The curves represent the citation relationship.

The literature search yielded 2,109 articles on melanoma immunotherapy published since 2010 (**Figure 1A**). In general, the publication number increased year by year, and rose significantly in 2015 and 2020. The total and median citation number of these 2109 articles was 137,686 and 11, respectively. Interestingly, papers published in 2015 made the greatest contribution to the total number of citations. In addition, papers published in 2010 had higher average citation number per paper (555.1) than papers published in other years. The publications which were the key nodes of the citation network were identified and presented (**Supplementary Figure S1**). The top 25 references which had strongest citation burst were identified and showed (**Supplementary Figure S2**).

The 100 top-papers were identified (**Supplementary Table S1**) and analyzed for bibliographic coupling (**Supplementary Figure S3**). Most of them were clinical studies (72 papers). Among them, 70 were related with ICB. These papers were cited as high as 86,393 times, which accounted for 62.7% of citations for all papers on melanoma immunotherapy. The median number of citations of the top-papers was 418.5 (range: 218–9824). The paper titled, “Improved survival with ipilimumab in patients with metastatic melanoma” by Hodi et al., was the most cited paper (9,824) and had the highest average citations per year (818.7) (22). The paper titled, “Tertiary lymphoid structures improve immunotherapy and survival in melanoma” by Jonsson et al. (2020), was latest among the top-papers (23). *New England Journal of Medicine* (*N. Engl. J. Med.*) published all the ten most cited papers (**Table 1**). Among these ten papers, nine were randomized trials regarding ICB, the other explored prognosis factors in patients treated with ipilimumab.

**Table 1:** The 10 most cited papers in melanoma immunotherapy between 2010 and 2022a^a^.

Rank	Title	Corresponding Author	Year	Total citations	Average citations per year (rank)
1	Improved Survival with Ipilimumab in Patients with Metastatic Melanoma	Hodi, FS	2010	9824	818.7 (1)
2	Combined Nivolumab and Ipilimumab or Monotherapy in Untreated Melanoma	Hodi, FS	2015	4953	707.6 (2)
3	Nivolumab in Previously Untreated Melanoma without BRAF Mutation	Robert, C	2015	3549	507 (3)
4	Pembrolizumab versus Ipilimumab in Advanced Melanoma	Robert, C	2015	3506	500.9 (4)
5	Ipilimumab plus Dacarbazine for Previously Untreated Metastatic Melanoma	Wolchok, JD	2011	3113	283 (10)
6	Nivolumab plus Ipilimumab in Advanced Melanoma	Wolchok, JD	2013	2940	326.7 (9)
7	Genetic Basis for Clinical Response to CTLA-4 Blockade in Melanoma	Chan, TA	2014	2614	326.8 (8)
8	Safety and Tumor Responses with Lambrolizumab (Anti-PD-1) in Melanoma	Ribas, A	2013	2496	277.3 (11)
9	Nivolumab and Ipilimumab versus Ipilimumab in Untreated Melanoma	Hodi, FS	2015	1856	265.1 (14)
10	Overall Survival with Combined Nivolumab and Ipilimumab in Advanced Melanoma	Wolchok, JD	2017	1840	368 (6)

a These ten papers were all published on N. Engl. J. Med.

### 3.1 Journals

**Figure 2:**
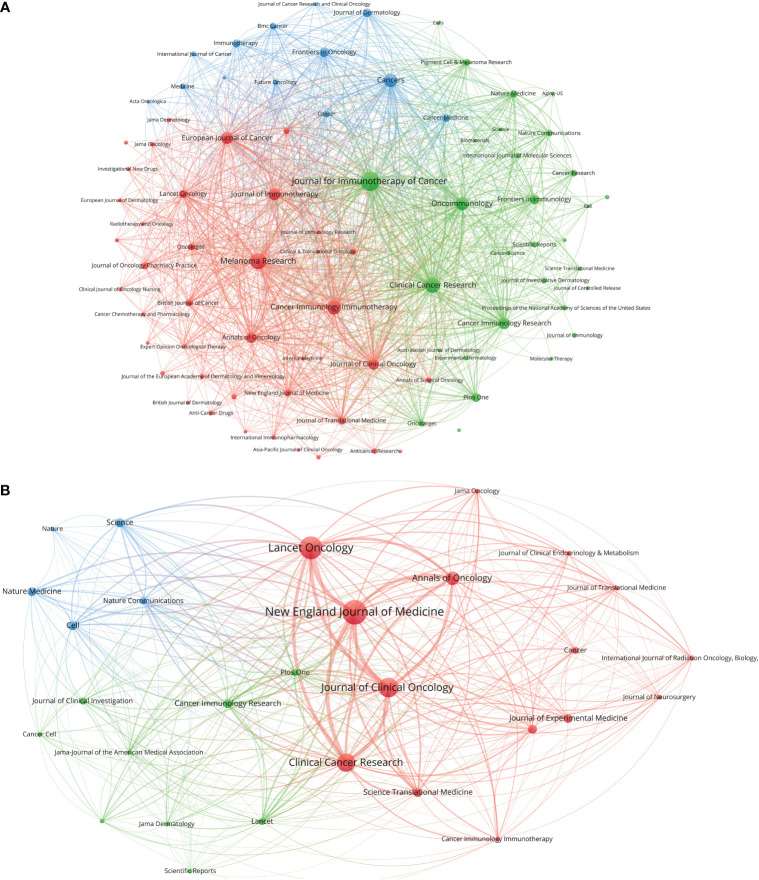
**(A)** Bibliographic coupling of journals with at least five papers related to melanoma immunotherapy. **(B)** Bibliographic coupling of journals with top-papers related to melanoma immunotherapy. The circle size represents the number of papers. The breadth of the curves represents the connection strength. The journals in the same color are of similar research areas.

The 2,109 articles were published in a total of 465 journals. *Journal for Immunotherapy of Cancer* published most papers (133 papers), followed by *Melanoma Research* (95 papers) and *Clinical Cancer Research* (*Clin. Cancer Res.*) (80 papers) (**Figure 1B**). The top 10 productive journals are listed in **Table 2**. Among these 10 journals, the *Journal of Clinical Oncology* had the highest average citations per paper (192.7), followed by *Clin. Cancer Res.* (109.4) and *OncoImmunology* (50.8). The top 10 journals with the most citations per paper per year are shown in **Figure 1C** and **Table 3**. Only 17 papers were published in the *N. Engl. J. Med.*, but they were cited as high as 42,682 time; this accounted for 31.0% of the citations of all papers on melanoma immunotherapy (average citation per paper: 2,510.7; citation per paper per year: 356.9). Among these 10 highly influential journals, the *Lancet Oncology* published most papers (25 papers). The inter-disciplinary citation dual-map overlay of journals related to melanoma immunotherapy were analyzed and presented (**Figure 1D**). The left nodes represented citing journals and the right nodes represented cited journals. The curves indicated the citation relationships. This mapping identified three colored primary citation pathways, implying that publications in molecular/biology/genetics were mainly cited by publications in molecular/biology/immunology or medicine/medical/clinical disciplines, while publications in health/nursing/medicine were primarily cited by publications in medicine/medical/clinical disciplines. The citation network of journals which published papers on melanoma immunotherapy is shown in **Figure 2A**.

**Table 2:** The top 10 productive journals in melanoma immunotherapy between 2010 and 2022.

Journals with most papers	Paper number	Total citation	Citation per paper	Citation per paper per year^a^	H-index	G-index	IF (2020)
J. Immunother. Cancer	133	3038	22.8	6.5	28	50	13.75
Melanoma Res.	95	1831	19.3	3.7	24	39	3.60
Clin. Cancer Res.	80	8753	109.4	17.5	43	78	12.53
Cancer Immunol. Immunother.	71	2145	30.2	5.6	25	45	6.97
Cancers	65	1891	29. 1	2.7	26	14	6.64
OncoImmunology	62	3149	50.8	7	28	42	8.11
J. Immunother.	52	294	5.7	4.4	10	37	4.46
Eur. J. Cancer	50	1489	29. 8	7	23	38	9.16
Cancer Immunol. Res.	47	1455	31.0	11.5	23	47	11.15
J. Clin. Oncol.	40	7709	192.7	34.2	30	40	44.54

a Papers published in 2022 were not included for calculating citation per paper per year.

**Table 3:** The top 10 journals with most citations per paper per year in melanoma immunotherapy between 2010 and 2022.

Journals with most papers	Paper number	Top-Paper number	TPR (2010-2020)^a^	Total citation	Citation per paper	Citation per paper per year^b^	Local citation^c^	IF (2020)
N. Engl. J. Med.	17	16	100.0%	42682	2510.7	356.9	6181	91.25
Science	6	3	100.0%	4520	753.3	202	1180	47.73
Nature	3	1	33.3%	751	250.3	112.7	1566	49.96
Lancet	4	2	50.0%	2171	542.8	102.3	690	79.32
Cell	5	3	60.0%	2481	496.2	100.1	968	41.58
Lancet Oncol.	25	14	70.0%	9784	391.4	72.8	2050	41.32
J. Exp. Med.	3	3	100.0%	2262	754	71	894	14.31
JAMA	2	1	50.0%	710	355	57.8	234	56.27
Sci. Transl. Med.	7	3	42.9%	2160	308.6	45.9	405	17.99
JAMA Oncol.	8	1	16.7%	1085	135.6	44.1	450	31.78

a Percentage of top-cited papers among all papers in a journal. The time span was from 2010 to 2020 (the year of publication of the most recent top-cited paper.

b Papers published in 2022 were not included for calculating citation per paper per year.

c Citation number in the current dataset (papers in melanoma immunotherapy between 2010 and 2022).

The 100 top-papers are listed in **Supplementary Table S1** and were published in 29 journals (**Supplementary Table S2**). The citation network of these journals is shown in **Figure 2B**. The *N. Engl. J. Med.* published most top-papers (16 papers). The TPR of the *N. Engl. J. Med.*, *Science*, and *Journal of Experimental Medicine* was 100%. Except *Journal of Translational Medicine* and *Cancer Immunology Immunotherapy*, all the other 27 journals had a TPR of >5%. Thus, the authors considered these journals as major journals on melanoma immunotherapy. A total of 118 articles published in major journals between 2020 and 2022 were identified (**Supplementary Table S2**). Among the major journals, *Clin. Cancer Res.* published the most papers (21 papers) since 2020.

### 3.2 Countries

**Figure 3:**
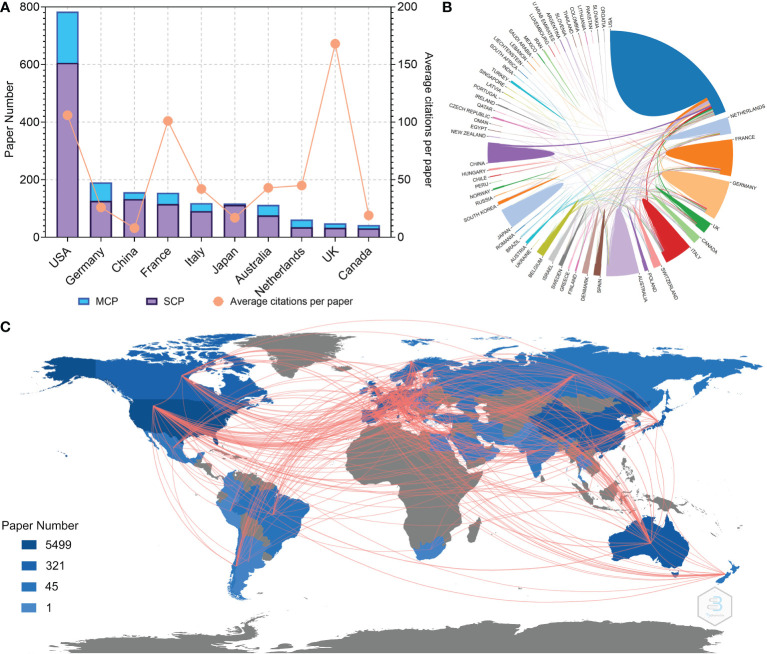
**(A)** Paper number and average citations of corresponding authors’ countries. MCP, multiple-country publications; SCP, single-country publications. **(B)** Network mapping of international collaboration. **(C)** Visualization world map of paper number and collaboration relationship.

**Figure 4:**
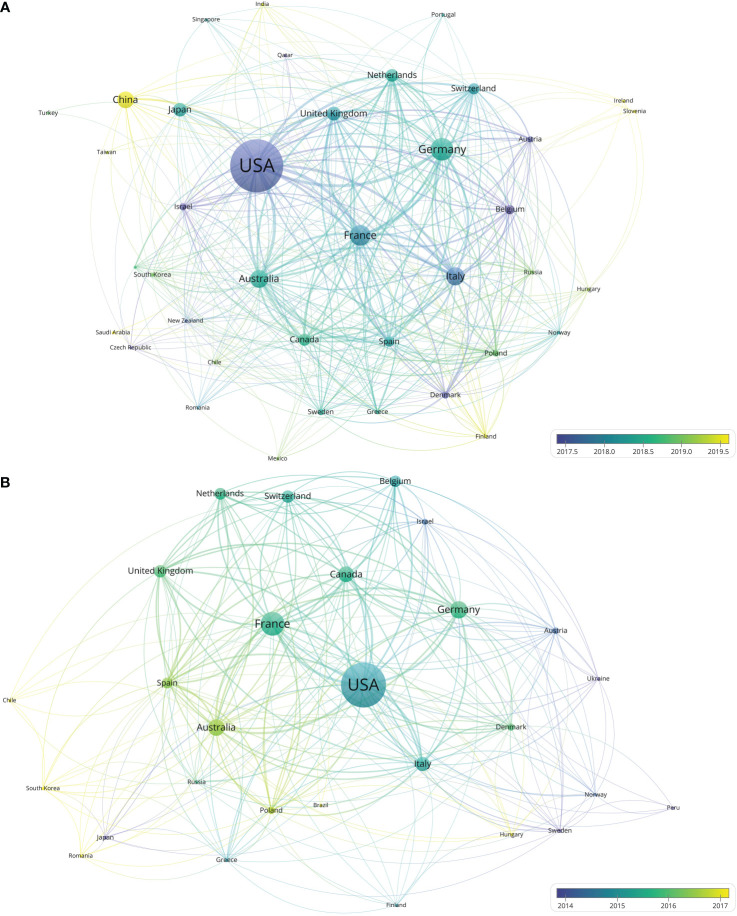
**(A)** Network visualization of countries with at least five papers related to melanoma immunotherapy. **(B)** Network visualization of countries with top-papers related to melanoma immunotherapy. The circle size represents the number of papers. The breadth of the curves represents the connection strength.

The authors and corresponding authors of these papers represented 59 and 47 countries/regions, respectively (**Table 4**). The USA took part in most papers (corresponding authors of 784 papers, **Figure 3A**). Averagely, papers with corresponding authors from the United Kingdom were most cited (168 citations per paper). Most studies were conducted by authors from single countries. The collaboration relationship between countries/regions is depicted in **Figures 3B, C**, **4A**. Several developed countries dominated collaborative research relationships, and contributed much more studies than others. Papers authored by researcher of Asian countries were mostly published in the recent few years.

**Table 4:** The top 10 productive countries of corresponding authors of papers in melanoma immunotherapy between 2010 and 2022.

Countries	Paper number	Percentage (N/2109)	Total citation	Citation per paper	Top-paper number^a^	Multiple-country top-paper rate^b^
USA	784	37.24%	83240	106	67	52.2%
Germany	191	9.07%	5137	26.90	3	100%
China	157	7.46%	1404	8	0	/
France	155	7.36%	15701	101.30	11	72.7%
Italy	119	5.65%	5088	42.76	4	50.0%
Japan	117	5.56%	2009	17.17	0	/
Australia	113	5.37%	322	29.27	4	75.0%
Netherlands	62	2.95%	2804	45.23	3	100%
United Kingdom	49	2.34%	8265	168.67	4	100%
Canada	43	2.04%	836	19.44	0	/

a Besides the countries mentioned above, corresponding authors from Switzerland, Israel, and Sweden contributed two, one, and one top-papers, respectively.

b Percentage of multiple-country top-papers among all top-papers of a country.

/, Not Applicable.

Twenty-nine countries contributed to the 100 top-papers. The network visualization maps for collaborations between these countries is shown in **Figure 4B**. The corresponding authors were from only 10 countries; most of them (corresponding authors of 67 papers) were from the USA. Collaboration between countries was common in top-papers, as most studies were conducted by authors from multiple countries. Although China, Japan, and Canada were productive, no top-papers were contributed by corresponding authors from these countries.

### 3.3 Institutions

**Figure 5:**
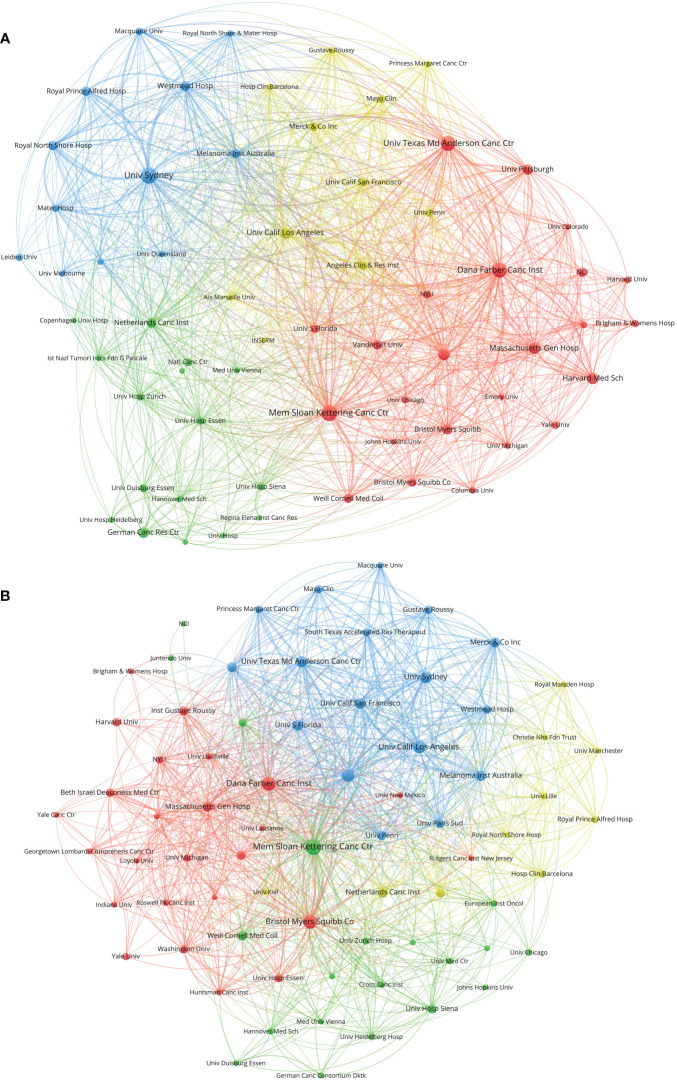
**(A)** Network visualization of institutions with at least 20 papers related to melanoma immunotherapy. **(B)** Network visualization of institutions with at least three top-papers related to melanoma immunotherapy. The circle size represents the number of papers. The breadth of the curves represents the connection strength. The institutions in the same color have stronger collaboration with each other.

A total of 3,098 institutions contributed to papers on melanoma immunotherapy. A co-author collaboration and citation mapping of the institutions was constructed (**Figure 5A**). The University of Texas MD Anderson Cancer Center had performed most studies (320 papers) among the institutions, followed by the University of Sydney (278 papers) and Memorial Sloan-Kettering Cancer Center (240 papers) (**Table 5**); 7 of the top 10 productive institutions were from the USA. Authors from 422 institutions contributed to top-papers. A co-author collaboration and citation mapping of these institutions was constructed (**Figure 5B**); the Memorial Sloan-Kettering Cancer Center published most top-papers (68 papers).

**Table 5:** The top 10 productive institutions in melanoma immunotherapy between 2010 and 2022.

Institutions	Country	Paper number^a^	Percentage (N/2109, %)	Top-paper number	Top-paper number rank
Univ Texas MD Anderson Canc Ctr	USA	320	15.17%	37	4
Univ Sydney	Australia	278	13.18%	34	5
Mem Sloan Kettering Canc Ctr	USA	240	11.38%	68	1
Dana Farber Canc Inst	USA	195	9.25%	44	3
Univ Pittsburgh	USA	151	7.16%	17	11
H Lee Moffitt Canc Ctr and Res Inst	USA	143	6.78%	12	21
Harvard Med Sch	USA	139	6.59%	13	19
Univ Calif Los Angeles	USA	139	6.59%	45	2
Netherlands Canc Inst	Netherlands	136	6.45%	20	9
Ascierto	Italy	115	5.45%	15	15

a All papers were included, without limitation of corresponding author’s institutions.

### 3.4 Authors

**Figure 6:**
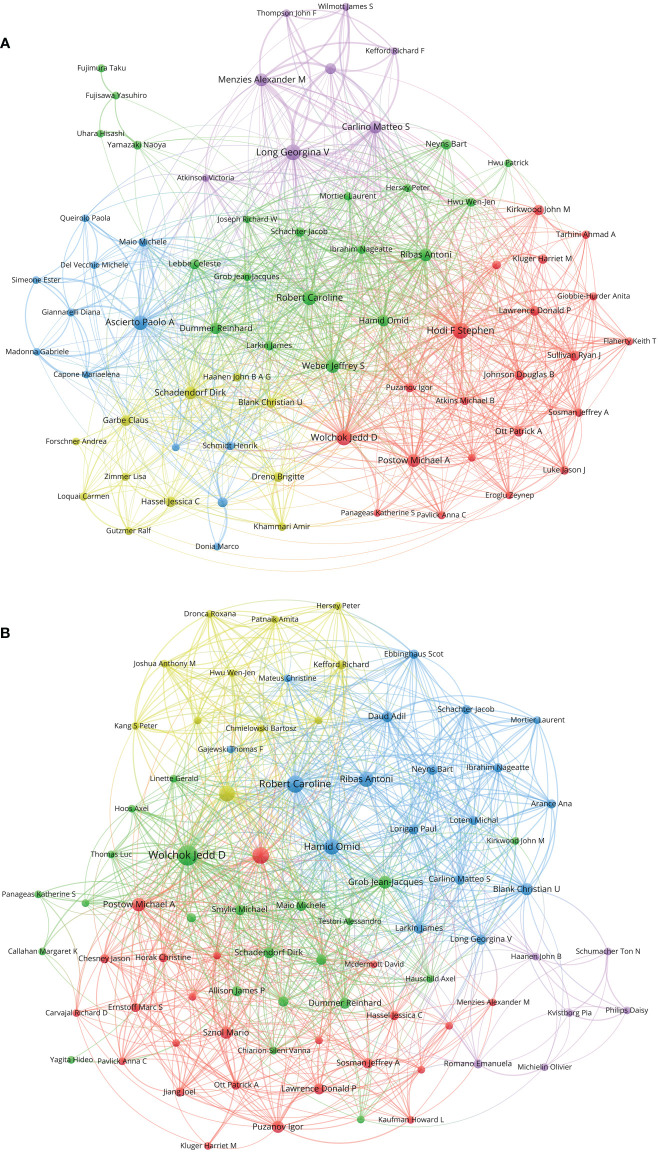
**(A)** Network visualization of authors with at least 15 papers related to melanoma immunotherapy. **(B)** Network visualization of authors with at least three top-papers related to melanoma immunotherapy. The circle size represents the number of papers. The breadth of the curves represents the connection strength. The authors in the same color have stronger collaboration with each other.

A total of 12,225 authors contributed to papers on melanoma immunotherapy. A co-author collaboration and citation mapping of the coauthors was constructed (**Figure 6A**). Hodi FS contributed most papers (77 papers) among the authors, followed by Ascierto PA (71 papers) and Robert C (64 papers). **Table 6** lists the authors of at least 40 papers. Among the authors, Wolchok JD had the highest H-index (24), average citations per paper (566.7 citations), local citations (1407 citations), and top-paper number (25 papers). A co-authorship analysis of the top-papers was showed in **Figure 6B**.

**Table 6:** Authors of at least 40 papers in melanoma immunotherapy between 2010 and 2022.

Name	Paper number	Total citation	H-index	G-index	Average citations per paper	Articles fractionalized^a^	Local citation^b^	Top-paper number
Hodi FS	77	33011	46	74	428.7	8.38	1353	20
Ascierto PA	71	17450	35	66	245.8	6.04	652	10
Robert C	64	34626	37	57	541.0	6.79	1266	21
Wolchok JD	59	33435	46	58	566.7	4.53	1407	25
Schadendorf D	53	23355	29	47	440.7	4.66	700	10
Carlino MS	52	11984	26	49	230.5	3.98	451	7
Long GV	51	20009	38	50	392.3	3.97	571	10
Menzies AM	47	3386	23	45	72.0	3.64	334	4
Ribas A	44	19622	32	41	446.0	2.51	1206	16
Hamid O	41	13235	32	40	322.8	3.10	1279	17
Larkin J	40	15690	22	37	392.3	4.08	298	9
Maio M	40	18835	32	40	470.9	2.40	745	16

a Articles Fractionalized = paper number/total number of authors of the papers.

b Citation number in the current dataset (papers in melanoma immunotherapy between 2010 and 2022).

### 3.5 Keywords and research trends

**Figure 7:**
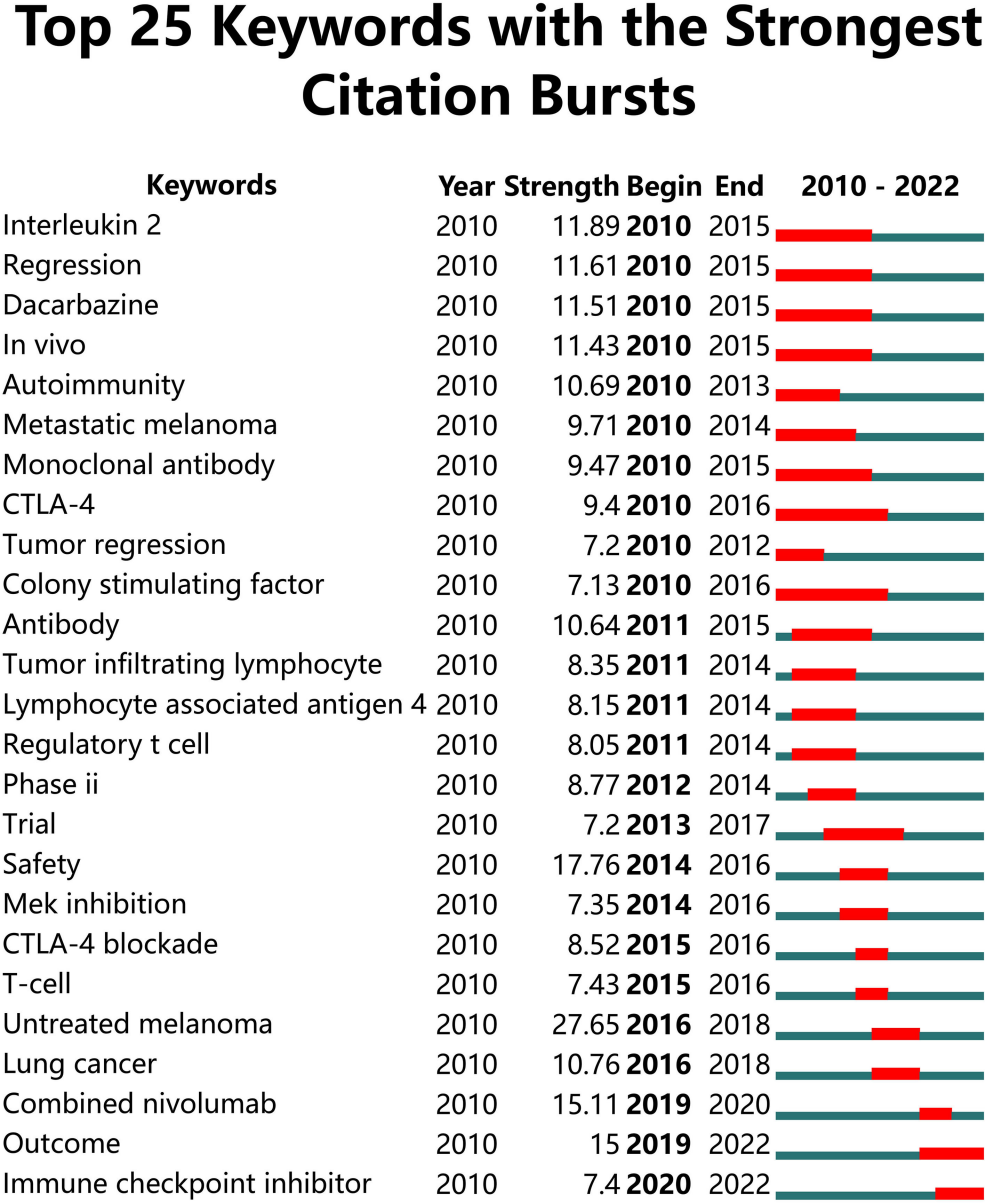
Top 25 keywords with the strongest citation bursts in papers on melanoma immunotherapy. The green line represents the timeline and the red line on the timeline represents the burst duration from the start year to the end year.

**Figure 8:**
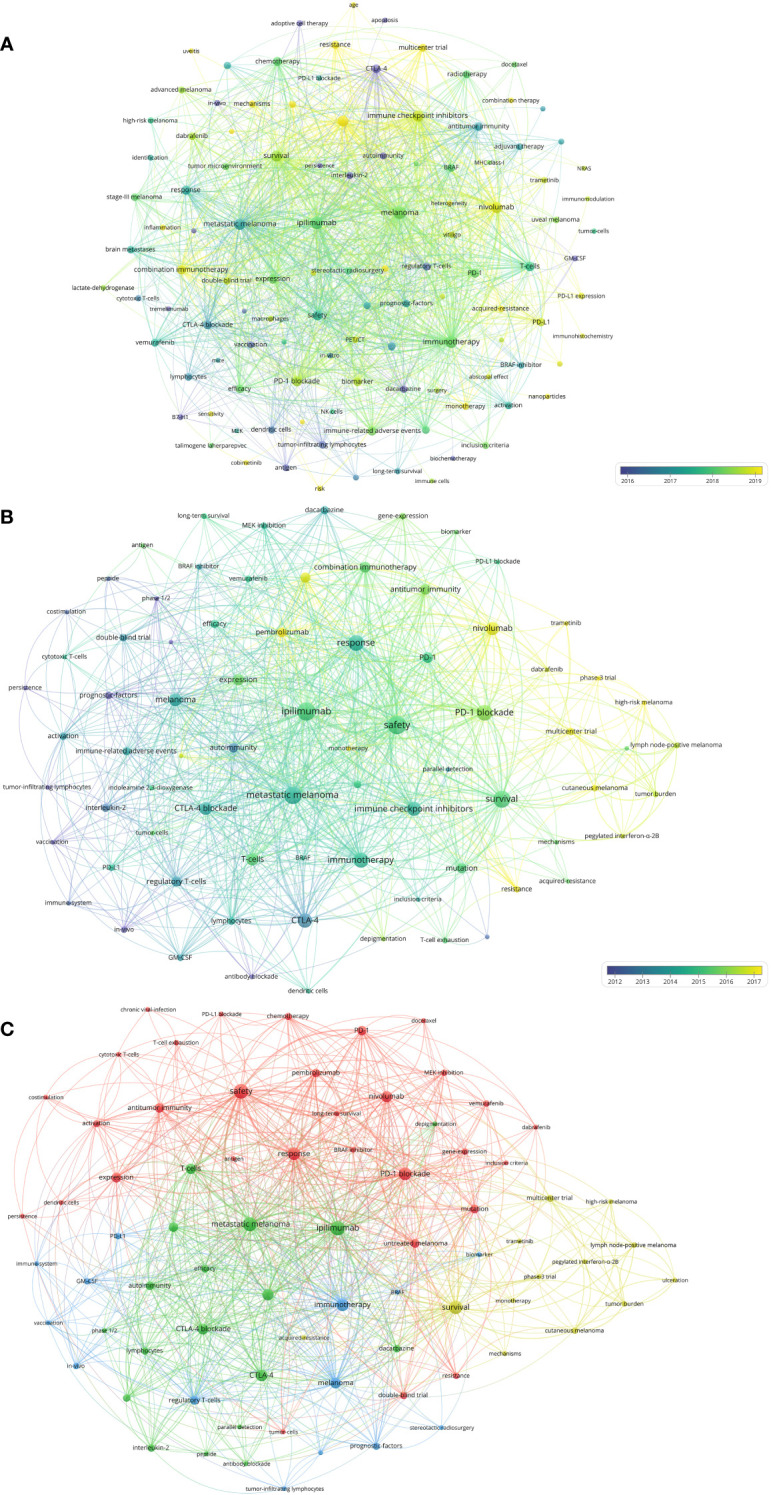
**(A)** Network visualization of keywords that occurred at least 10 times in the papers. **(B)** Network visualization of keywords that occurred at least twice in the top-papers. **(C)** Network visualization of keywords that occurred at least twice in papers published in major journals between 2020 and 2022. The circle size represents the number of papers. The breadth of the curves represents the connection strength.

The top twenty-five keywords which had strongest citation burst in papers on melanoma immunotherapy were identified (**Figure 7**). The co-occurrence and citation mapping of the keywords was conducted (**Figure 8A**). The top-occurred keywords included “ipilimumab”, “nivolumab”, “pembrolizumab”, “metastatic melanoma”, “survival”, “safety”, “response”, and “T-cell”. Recently utilized keywords included “acquired-resistance”, “multicenter trial”, “PD-L1”, “monotherapy”, inflammation”, “heterogeneity”, “biomarker”, and “combination immunotherapy”. The keyword co-occurrence analysis of the top-papers was conducted (**Figure 8B**). The newly arisen keywords of top-papers included “high-risk melanoma”, “dacarbazine”, “untreated-melanoma”, “lymph node-positive melanoma”, “regulatory T-cell”, “T-cell exhaustion”, and “BRAF”. The trends of keyword frequencies on melanoma immunotherapy between 2010 and 2022 are shown in **Supplementary Figure S4**.

The 118 melanoma immunotherapy articles published in the major journals between 2020 and 2022 were analyzed to conduct a keyword co-occurrence network (**Figure 8C**). The keywords which differed from those included in the previous analysis included “tumor mutational burden”, “stereotactic radiosurgery”, “tumor burden”, “GM-CSF”, and “MEK-inhibition”.

**Figure 9:**
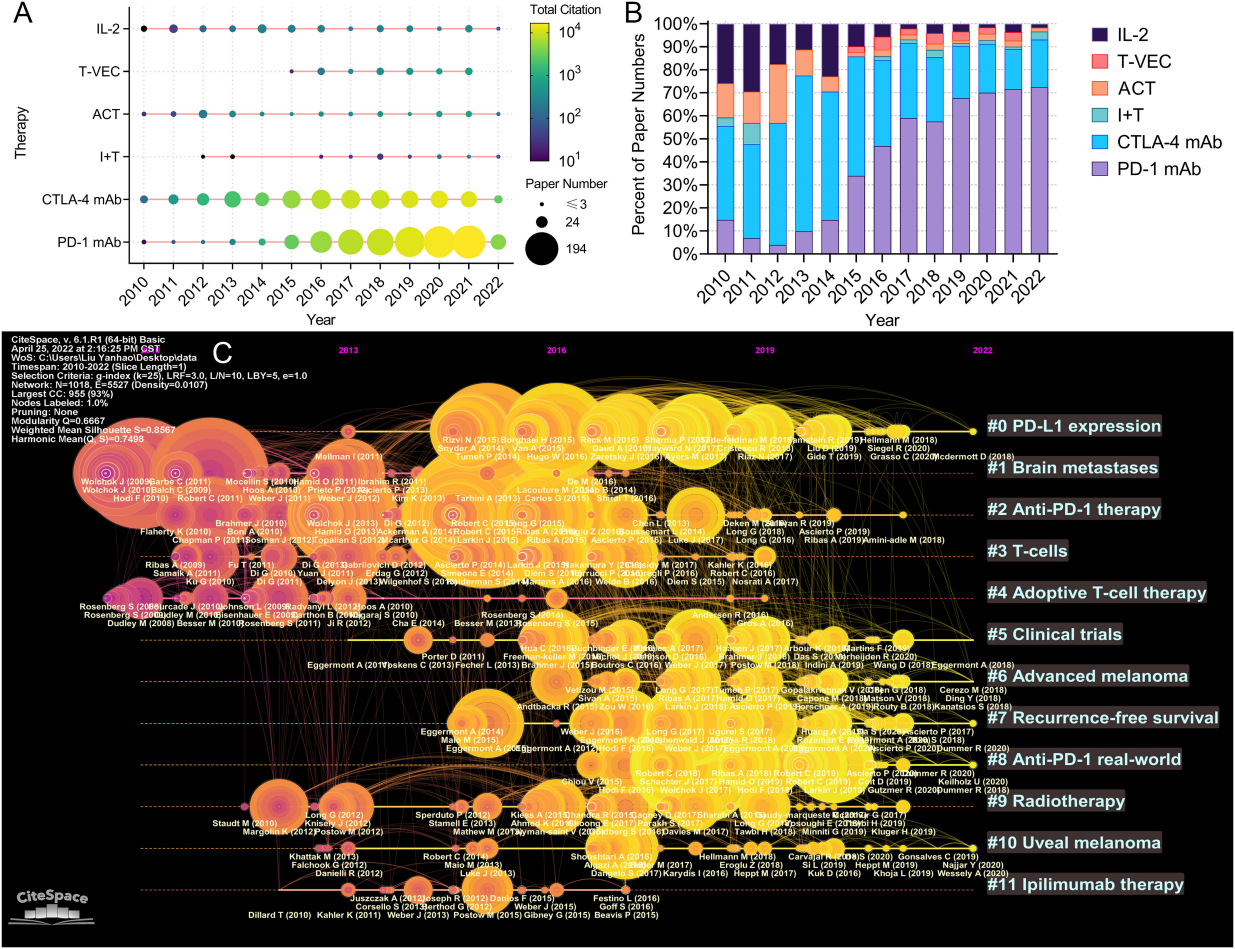
**(A)** Publication and citation number of papers evaluating different therapies. The node size represents the paper number and the color represents the average citations per paper. **(B)** The percentage of papers evaluating different therapies each year. **(C)** The timeline view for co-cited references related to melanoma immunotherapy. The node size represents the citation number of the reference. The curves between the nodes indicated co-citation relationships. Yellow nodes represent new papers and red nodes represent old ones.

The paper number and total citation per year of the six immunotherapies for melanoma are shown in **Figure 9A**. Paper numbers for CTLA-4 blockade increased between 2010 and 2015, while paper numbers for PD-1 blockade rose since 2015. Most papers on T-VEC and immunotherapy plus targeted therapy were published after 2015. The proportions of the papers published on the six immunotherapies each year are shown in **Figure 9B**. The proportion of papers on IL-2 and adoptive cell immunotherapy decreased continuously, while that on PD-1 blockade steadily increased. The papers on CTLA-4 blockade accounted for the highest proportion between 2010 and 2015. A timeline graph of co-cited references related to melanoma immunotherapy is shown in **Figure 9C**. The clusters with yellow and large nodes included numerous new publications, demonstrating the topics of these clusters to be research hotspots. According to the cluster labels and references in the clusters, the recent research hotspots included “tumor immune microenvironment”, “clinical research of anti-PD-1 immunotherapy”, “neoadjuvant and adjuvant immunotherapy”, and “radiotherapy combined with immunotherapy”.

## 4 Discussion

Immunotherapy, mainly represented by ICB, has rapidly become the mainstay of treatment for metastatic melanoma in recent years. Only a few years after the clinical application of CTLA-4 blockade, PD-1 blockers achieved even significantly superior outcome for metastatic melanoma (25, 26). In recent years, ICB has been proven to be beneficial as neoadjuvant and adjuvant treatment for high-risk resected melanoma (27–29). Additionally, other immunotherapy modalities such as ACT and T-VEC also showed clinical value in selected patients with melanoma (30, 31). Further research is underway to develop superior treatment strategies, predictive biomarkers, as well as management of immune-related adverse effects (irAEs).

### 4.1 CTLA-4 and PD-1 blockades

CTLA-4 and PD-1 are mainly expressed on the cytomembrane of immune cells; their primary function is to keep immune tolerance and restrict inflammation in normal tissues. Their ligands, B7 and PD-L1, are normally expressed on the cytomembrane of antigen-presenting cells and sometimes on tumor cells (32). The PD-1:PD-L1 and CTLA-4:B7 interactions suppress the activity of T-cells, thus regulating cytokine secretion and affecting immune-cell multiplication and phenotype (33). Therefore, PD-L1 and B7 expressed on tumor cells contribute to immune escape by suppressing cytotoxicity. By breaking the interactions, CTLA-4/PD-1/PD-L1 blockades up-regulate T-cell activity and strengthen anti-tumor immune response (34). Furthermore, the recently revealed role of PD-L1:B7-1 cis-interaction advised that blocking both PD-1 and CTLA-4 may create synergistic effects, thus potentially strengthening T-cell activation (33).

#### 4.1.1 ICB for metastatic melanoma

CTLA-4 blockade used to be the most popular treatment for metastatic melanoma between 2010 and 2015; however, PD-1 blockade has taken over since 2015. In 2010, Hodi et al. reported ipilimumab for metastatic melanoma improved OS in the first phase 3 trial (22). A pooled analysis demonstrated that 4,846 melanoma patients who received ipilimumab achieved a 3-year OS rate of 21%. The survivorship curve started to flatten out since approximately the third year, supporting the durability of responses to ipilimumab (35). In 2015, two phase 3 trials (CheckMate 067 and KEYNOTE-006) demonstrated significantly superior efficacy and tolerability of PD-1 blockades (nivolumab and pembrolizumab) to ipilimumab (25, 26). In addition, the results of KEYNOTE-006 trial showed that the 5-year OS rates of patients who received pembrolizumab and ipilimumab were 38.7% and 31.0%, respectively; grade 3-4 irAEs were observed in 17% and 20% of the patients, respectively (36).

Many trials evaluated dual-ICB to further improve the efficacy. In the CheckMate 067 trial, patients in three groups received nivolumab combined with ipilimumab, single-agent nivolumab, and single-agent ipilimumab, respectively. The dual-ICB group achieved a median OS that extended up to 72.1 months, which was much longer than that in the nivolumab (36.9 months) and ipilimumab (19.9 months) groups (37). However, dual-ICB also resulted in a considerably higher risk of grade 3/4 irAEs (55.0% versus 16.3% and 27.3%), that could induce treatment discontinuation (11). Among patients with ICB therapy discontinuation in the three groups, the median ICB-free times were 27.6, 2.3, and 1.9 months, respectively (37). Remarkably, irAEs seemed to be related to potent and durable immune responses, as the outcome of patients with ICB therapy discontinuation due to irAEs was comparable with that of all patients (11).

The optimal dosing strategy and patient selection for dual-ICB were highly focused. The KETNOTE-029 phase 2 trial aimed to optimize the dosing schedule of dual-ICB. Patients who received pembrolizumab (200 mg Q3W) combined with ipilimumab (50 mg Q6W) experienced superior toxicity and similar PFS compared with those who received pembrolizumab (200 mg Q3W) combined with ipilimumab (100 mg Q12W) (38). The ADAPT-IT phase 2 trial evaluated adaptive dosing of dual-ICB based on early and interim radiographic assessments. After two cycles of dual-ICB, patients who achieved a protocol-defined early favorable antitumor effect were transferred to nivolumab monotherapy, while other patients received two additional cycles of dual-ICB and were subsequently transferred to nivolumab monotherapy. The ORR was 58%, and 57% of the patients had grade 3-5 toxicity (39). Moreover, the CheckMate 204 trial also demonstrated the superiority of dual-ICB to nivolumab for patients with active brain metastatic melanoma (40). A recently published study retrospectively evaluated the efficacy of dual-ICB in older patients with metastatic melanoma. The results suggested that patients >= 65 years received similar benefit and toxicity in comparison to their younger counterparts (41). However, another study evaluated ICB for elderly patients who were fit or frail, and suggested that while frailty was not associated with severe irAEs, it was an indicator of adverse sequelae associated with irAEs, such as hospital admission (42).

#### 4.1.2 Adjuvant ICB therapy

Adjuvant ICB therapy might lower the recurrent rate in high-risk patients with resected melanoma. In 2015, the EORTC 18071 phase 3 trial demonstrated adjuvant ipilimumab therapy prolonged the recurrence-free survival (RFS) for resected stage III melanoma (28). Only 2 years later, the CheckMate-238 phase 3 trial demonstrated adjuvant nivolumab therapy offered longer disease-free survival and lower toxicity than ipilimumab for patients with advanced melanoma (27). In 2020, the IMMUNED trial compared adjuvant dual-ICB with single-agent therapy for metastatic melanoma patients. Dual-ICB yielded higher 2-year RFS rate (70% versus 42%), but resulted in higher grade 3/4 trAEs incidence (71% versus 27%) compared with single agent therapy (43). In 2022, the first interim analysis of the KEYNOTE-716 phase 3 trial suggested that adjuvant pembrolizumab therapy for up to 1 year for stage IIB or IIC melanoma significant reduced the risk of recurrence or death, with a manageable toxicity (44).

#### 4.1.3 Neoadjuvant ICB therapy

Given the success of adjuvant ICB treatment, there has been a considerable focus on neoadjuvant ICB treatment for patients with resectable melanoma in recent years. In 2018, a phase 2 trial suggested that compare with adjuvant treatment, neoadjuvant dual-ICB expands greater tumor-resident T-cell clones (45). Another phase 2 randomized trial compared the ORR, pathologic complete response (PCR) rates, and trAEs of nivolumab monotherapy and dual-ICB as neoadjuvant therapy. Dual-ICB resulted in potent responses (ORR: 73%; PCR: 45%), but was associated with unreliable safety (grade 3 trAEs: 73%); conversely, single-agent nivolumab resulted in moderate responses (ORR, 25%; PCR, 25%) and superior safety (grade 3 trAEs, 8%) (29). In order to determine the optimal dosing strategy of dual-ICB, the OpACIN-neo phase 2 randomized trial included three groups of patients with locally advanced melanoma receiving different dosing strategies. Patients who received nivolumab (3 mg/kg) combined with ipilimumab (1 mg/kg) for two cycles achieved both high pathologic response rate (77%) and satisfactory safety (grade 3/4 irAEs, 0%) (46). The recently published PRADO trial evaluated personalized response-directed surgery and adjuvant therapy after neoadjuvant dual-ICB in high-risk stage III melanoma. In this trial, patients achieved major pathological response (MPR) could omit lymph node dissection and adjuvant therapy; patients achieved partial pathologic response (pPR) only received lymph node dissection; and patients with pathologic non-response (pNR) received lymph node dissection and adjuvant systemic therapy. The 24-month RFS and distant metastasis-free survival rates were 93% and 98% in patients with MPR, 64% and 64% in patients with pPR, and 71% and 76% in patients with pNR, respectively (24). Phase III trials regarding neoadjuvant therapy for melanoma are ongoing.

#### 4.1.4 Biomarkers for ICBs

Biomarkers and predictive models are essential for treatment decision-making. An analysis of the OpACIN and OpACIN-neo trial revealed that high TMB and high interferon–related gene expression signature score were related to great efficacy in patients who received dual-ICB (47). In addition, the data of the COMBI-I trial showed that low T cell-inflamed gene expression signature or high immunosuppressive tumor microenvironment were associated with short PFS (48). A genome-wide expression analysis suggested that the multipotency and differentiation status of melanoma can determine ICB benefit (49). Furthermore, the baseline maximal glycolytic activity and gut microbiome were proved to be related with the response after ICB therapy (50, 51).

In recent years, PD-1 blockade has been a standard treatment for metastatic melanoma; dual-ICB results in improved efficacy with higher toxicity. Dual-ICB also shows superior efficacy as adjuvant or neoadjuvant therapy compared to nivolumab monotherapy, but the dosing schedule needs to be optimized to minimize toxicity. Additionally, in view of comparable disease-free survival, the treatment decision-making for patients suitable for both adjuvant immunotherapy and targeted therapy remains challenging (52). Currently, multiple biomarkers including clinical, genomic, transcriptomic, gut microbiome, and radiomic features have been proven to be related to responses to ICB treatment (53). ​However, further exploration of biomarkers and mechanisms at the genomic and transcriptome levels remains essential. Moreover, given the high incidence of severe IAEs with dual-ICB, a more robust predictive model incorporating multiple biomarkers and clinical evidence is warranted to identify subgroups of patients who may benefit from dual-ICB.

### 4.2 Combining immunotherapy with targeted therapy

Patients with BRAF gene altered melanoma who received BRAF and MEK inhibitors (BRAFi/MEKi) may achieve high response rates; however, immunotherapy usually results in durable responses (8). The combination of these treatments has been a research hotspot in recent years. In 2013, initial studies demonstrated grade 3 hepatotoxicity with ipilimumab plus vemurafenib (54). Several early-phase clinical trials evaluated PD-1 (or PD-L1) plus BRAFi/MEKi triplet therapy and reported high response rates and incidences of grade 3/4 trAEs (55–58). In 2020, the results of the IMspire150 trial showed that adding atezolizumab to BRAFi/MEKi in patients with BRAF(V600)-mutated advanced melanoma resulted in improved PFS with acceptable tolerance (59). PD-L1 level, lactate dehydrogenase level, interferon-γ, and TMB were primary predictors of outcomes (60). Some trials evaluated immunotherapy combined with targeted therapies other than BRAFi/MEKi. The PIVOT-02 trial reported that first-line bempegaldesleukin plus nivolumab for metastatic melanoma provided improved PFS and acceptable toxicity (61). A phase 2 trial reported that ceralasertib plus durvalumab showed favorable efficacy in patients with metastatic melanoma which progressed after PD-1 blockade therapy (62). The recently published TRICOTEL phase 2 trial reported that atezolizumab plus vemurafenib and cobimetinib provided promising intracranial anti-tumor activity and acceptable toxicity in patients with BRAF(V600)-mutated melanoma (63).

Immunotherapy in combination with targeted therapy results in a potent response, but is usually associated with unsatisfactory toxicity. In combination with targeted therapy, PD-L1 blockades appear to be more tolerable than PD-1 blockades. New targeted therapies other than BRAFi/MEKi can improve efficacy and safety, but high-quality clinical evidence is lacking. Further research is needed to determine the optimal strategy of drug selection and combination.

### 4.3 Other immunotherapy

Adoptive cell immunotherapy, with a history of being used to treat melanoma for approximately two decades, could augment tumor antigen-specific immunity *in vivo* by *in vitro* selection and proliferation of tumor antigen-specific T-cell clones (64). ACT was usually used as a salvage therapy for refractory metastatic melanoma; a proportion of patients could achieve durable responses after ACT (65). Lymphodepletion, which was necessary before cell return infusion, limited the application of ACT. A pooled-analysis compared treatment efficacy between three trials which employed different lymphodepleting regimens (chemotherapy alone, or with total body irradiation to a dose of 2 or 12 Gy). Patients who received 12 Gy irradiation achieved the highest ORR (72%) (66). Research on ACT has progressed slowly in recent years. Despite considerable advances in ICB therapy, the application of ACT for melanoma is restricted. Although ACT was commonly used in patients who failed after ICB, several recent studies suggested that prior anti-PD-1 therapy may potentially impair the efficacy of ACT (67, 68).

T-VEC is an engineered virus that can proliferate and synthesize granulocyte macrophage colony-stimulating factors specifically in melanoma cells to systemically promote antitumor immunity (31). T-VEC could be injected into melanoma in skin or accessible lymph nodes. Uninjected lesions may respond to T-VEC *via* a systemic immune priming effect (8). A phase 3 randomized trial reported T-VEC in treating advanced melanoma resulted in an ORR of 26.4% and a grade 3/4 trAEs incidence of 2% (31). A phase 2 randomized trial compared T-VEC plus ipilimumab with ipilimumab alone for patients with advanced melanoma. T-VEC plus ipilimumab yielded a apparently higher ORR (39% versus 18%) and a slightly higher occurrence rate of grade 3/4 trAEs (45% versus 35%) than ipilimumab (69). Given the favorable tolerance and proven immune priming effect, neoadjuvant T-VEC therapy was a considerable choice. A phase 2 randomized trial reported that neoadjuvant T-VEC reduced the recurrence risk in patients with resectable melanoma by approximately 25% (70).

Novel immunotherapy agents is warranted to conquer the innate resistance to anti-PD-1/PD-L1 therapy. In 2019, the ECHO-301/KEYNOTE-252 randomized trial evaluated epacadostat, an indoleamine 2,3-dioxygenase inhibitor, plus pembrolizumab for untreated advanced melanoma. However, adding epacadostat to pembrolizumab monotherapy improved neither PFS nor OS (71). In 2021, a phase 1/2 trial evaluated a therapeutic vaccine (IO102/IO103) and PD-L1 in combination with nivolumab; the combination showed favorable antitumor activity among metastatic melanoma patients (72). A phase 2/3 randomized trial (RELATIVITY-047) evaluated relatlimab (a lymphocyte-activation gene 3 blockade) combined with nivolumab as 1st-line therapy for advanced melanoma. Relatlimab plus nivolumab offered superior PFS with slightly increased toxicity compared to nivolumab monotherapy (73). Moreover, a recent trial suggested that neoadjuvant relatlimab plus nivolumab was effective and safe in patients with resectable melanoma (74).

### 4.4 Journals, countries, institutions, and authors

The *Journal for ImmunoTherapy of Cancer* is the most productive journal regarding melanoma immunotherapy. In this research area, clinical studies, especially randomized trials regarding ICB, were more likely to be highly cited. Therefore, *N. Engl. J. Med.*, whose majority of published papers were high-quality randomized trials regarding ICB, incontestably become the most influential of the journals. Notably, there was no duplicate journal between the top 10 productive journals and the top 10 journals with the most citations per paper per year. Twenty-seven journals were identified as major journals in this area; this indicates that papers published in these journals are more likely to be influential. Interestingly, some comprehensive journals, including the *N. Engl. J. Med.*, *Lancet Oncology*, and *Journal of Clinical Oncology* are considerably more influential than those focused on “melanoma” or “dermatology.” This may be due to the fact that these comprehensive journals have higher impact factors, thus influencing the decision of the authors.

Authors and institutions from the USA have made the greatest contribution to melanoma immunotherapy. Broad cooperation was common in a large number of studies in melanoma immunotherapy, as most prominent studies were sponsored by transnational pharmaceutical companies. Recent published phase 3 trials have reported data from developing countries/regions. Nevertheless, authors and data from Africa are lacking. The most prolific authors of top-papers were Wolchok JD, Robert C, and Hodi FS.

### 4.5 Research trends, status, and hotspots

The major review methods include systematic literature review, meta-analysis, and bibliometric analysis (13). Systematic literature review is suitable for qualitatively summarizing the advances on a specific research topic. However, it is limited by the knowledge and opinions of the authors, and cannot evaluate a large number of publications. Meta-analysis can summarize the evidence of multiple homogenous studies to address specific questions. Therefore, these two review methods are not able to present the status of the intellectual structure and emerging trends of an entire research area (13). Compared with systematic review and meta-analysis, this study is quantitative, comprehensive, and objective, with more than two thousand publications included. Based on the large dataset and rigorous analysis methods, the research trends, status, and keywords are demonstrated.

The keywords in the titles of the 2109 papers reflected the research trends. Between 2010 and 2022, CTLA-4 blockade has always been an important topic in melanoma, while PD-1 blockade has become more popular since 2016. Since 2019, PD-1 blockade has been evaluated in approximately 70% of melanoma immunotherapy papers each year. Papers related to IL-2 or ACT constitute a considerable share in melanoma immunotherapy, but the proportion has gradually decreased since 2013.

Currently, the research status is as follows: 1) single-agent PD-1 blockade has become the standard treatment for metastatic melanoma, 2) combining PD-1/PD-L1 blockade with CTLA-4 blockade or targeted therapy results in improved efficacy and increased toxicity, 3) neoadjuvant or adjuvant PD-1 blockade reduces the risk of recurrence; dual-ICB offers either better efficacy or increased toxicity, and 4) ACT is usually used as a salvage treatment for refractory metastatic melanoma, whereas T-VEC is usually administered as an adjunct to ICB.

The current research hotspots related to melanoma immunotherapy are as follows: 1) the optimal dosing schedule and patient selection for dual-ICB; 2) comprehensive and robust predictive biomarkers/models; 3) PD-L1 in combination with targeted therapy; 4) management of irAEs; 5) neoadjuvant and adjuvant immunotherapy; 6) overcoming inherent and acquired drug resistance; and 7) next generation immunotherapy.

The convincing results can help researchers understand the landscape of melanoma immunotherapy research. Based on these results, the authors suggest the most important research directions in the future include: 1) the optimal management strategies with the combination of existing treatments; 2) the robust predictive models for patient selection; 3) the tumor immune microenvironment and the mechanisms of immune-resistance; 4) novel immunotherapies to overcome resistance and further improve the prognosis. This research is beneficial in terms of research management, topic selection, and project funding.

### 4.6 Limitations

This study had certain limitations. First, it only included papers published since 2010, in which year the first phase 3 trial results on CTLA-4 blockade were published. The earlier trials and basic studies were therefore excluded. However, the limited time span of the papers essentially highlighted the research trends in the most recent years. Second, given the large volume of papers, the authors were unable to classify the findings by reading every paper. Therefore, more detailed analyses of the sub-divided area were not feasible. To overcome this limitation, the authors discussed the major modalities of immunotherapy to highlight the important advances. Third, although some basic studies directly related to immunotherapy were included, this study mainly focused on clinical issues. Thus, studies that were indirectly related to immunotherapy may have been omitted, and basic immunotherapy research was not discussed. Finally, the authors only used the Web of Science database to search for papers. Hence, publications in other databases may have not been included. This possibly introduce bias into the paper selection, result in error in citation analysis, and lead to the omission of important studies.

## 5 Conclusion

To the authors’ knowledge, this study is the first comprehensive and metrological bibliometric analysis of original research on melanoma immunotherapy. Based on the quantitative analysis of more than two thousand original articles, this study demonstrates the research trends and hotspots and the results are repeatable, reliable, and convincing. Moreover, this research is beneficial in terms of selecting research topics, selecting target journals for publication, finding potential collaborators, research management, and project funding. The authors suggest that the promising research directions in the future include: 1) optimal combination of existing treatments; 2) robust predictive models; 3) tumor immune microenvironment and immune-resistance mechanisms; 4) novel immunotherapies. This study can help researchers get a comprehensive picture of the research panorama, historical development, and recent hotspots in melanoma immunotherapy and provide inspiration for further research.

## Data availability statement

The raw data supporting the conclusions of this article will be made available by the authors, without undue reservation.

## Author contributions

XZ and YHL contributed to the study conception. YHL analyzed the data. YHL, LY, YJL, XC, SJ, HY, ZZ, LL, BQ, and YC contributed to the literature review. YHL wrote the manuscript. All authors contributed to the article and approved the submitted version.

## Supplementary material

Supplementary Figure 1 Historical direct citation network among the papers.

Supplementary Figure 2 Top 25 references with the strongest citation bursts in papers in melanoma immunotherapy. The green line indicates the timeline, and the intervals in which bursts were found are indicated by red sections on the timeline, indicating the start year, the end year, and the burst duration.

Supplementary Figure 3 Visualization of citation relationship between the papers with the strongest link strength.

Supplementary Figure 4 The trends of the keywords rank of melanoma immunotherapy from 2010 to 2022.

Supplementary Material S1 Detailed search strategy for the papers on melanoma immunotherapy.

Supplementary Table 1 The 100 most cited papers on melanoma immunotherapy.

Supplementary Table 2 The journals with top-cited papers on melanoma immunotherapy.

Supplementary Table 3 The papers on melanoma immunotherapy published in major journals since 2020.
