# Supplementary figures and images for: Research landscape and trends of melanoma immunotherapy: A bibliometric analysis
Source: Front Oncol. 2023 Jan 9;12:1024179. doi: 10.3389/fonc.2022.1024179 (PMC9868470; doi:10.3389/fonc.2022.1024179)

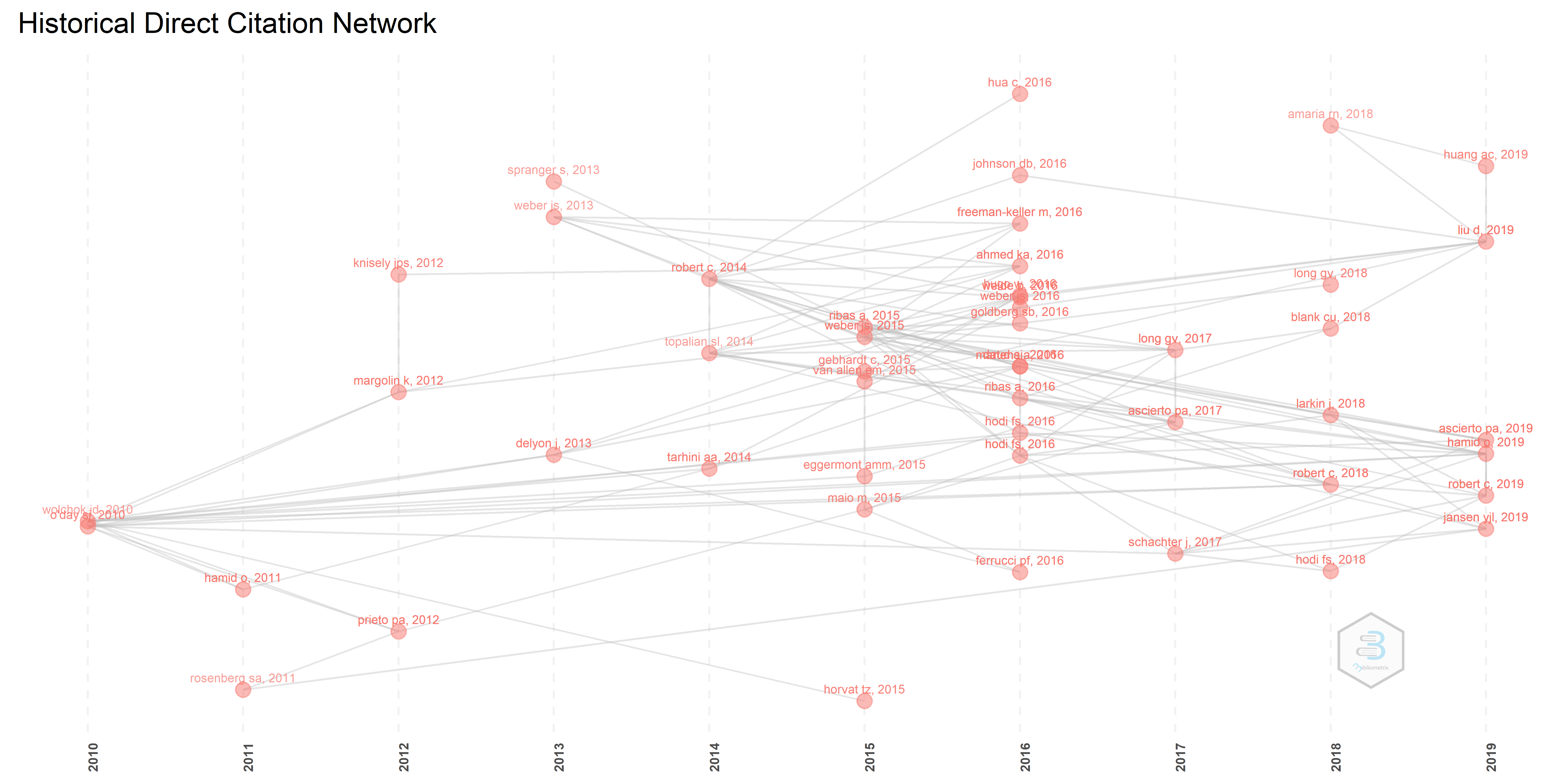

Supplement: Supplementary Figure 1 — Historical direct citation network among the papers. [file Image_1.png]

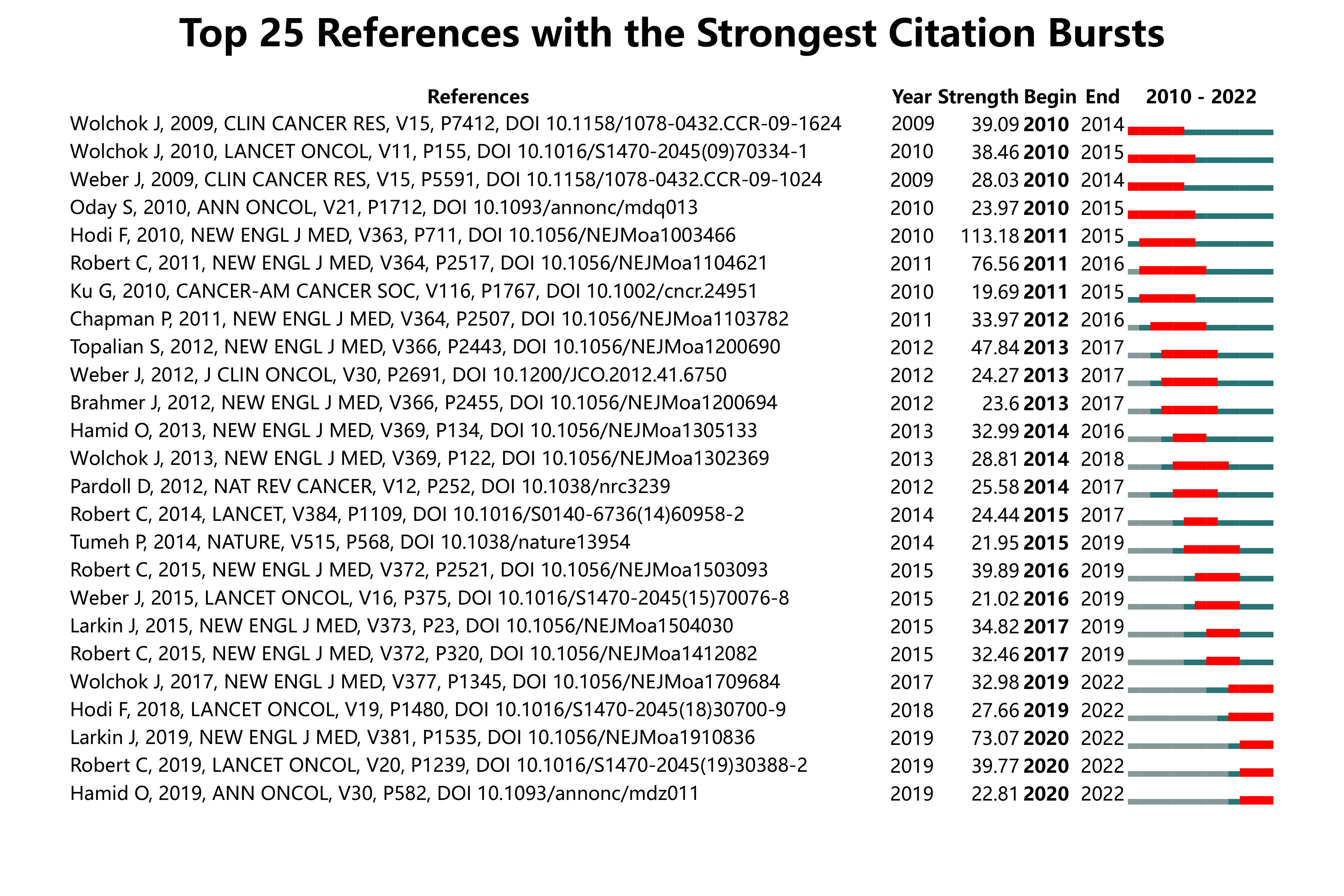

Supplement: Supplementary Figure 2 — Top 25 references with the strongest citation bursts in papers in melanoma immunotherapy. The green line indicates the timeline, and the intervals in which bursts were found are indicated by red sections on the timeline, indicating the start year, the end year, and the burst duration. [file Image_2.tif]

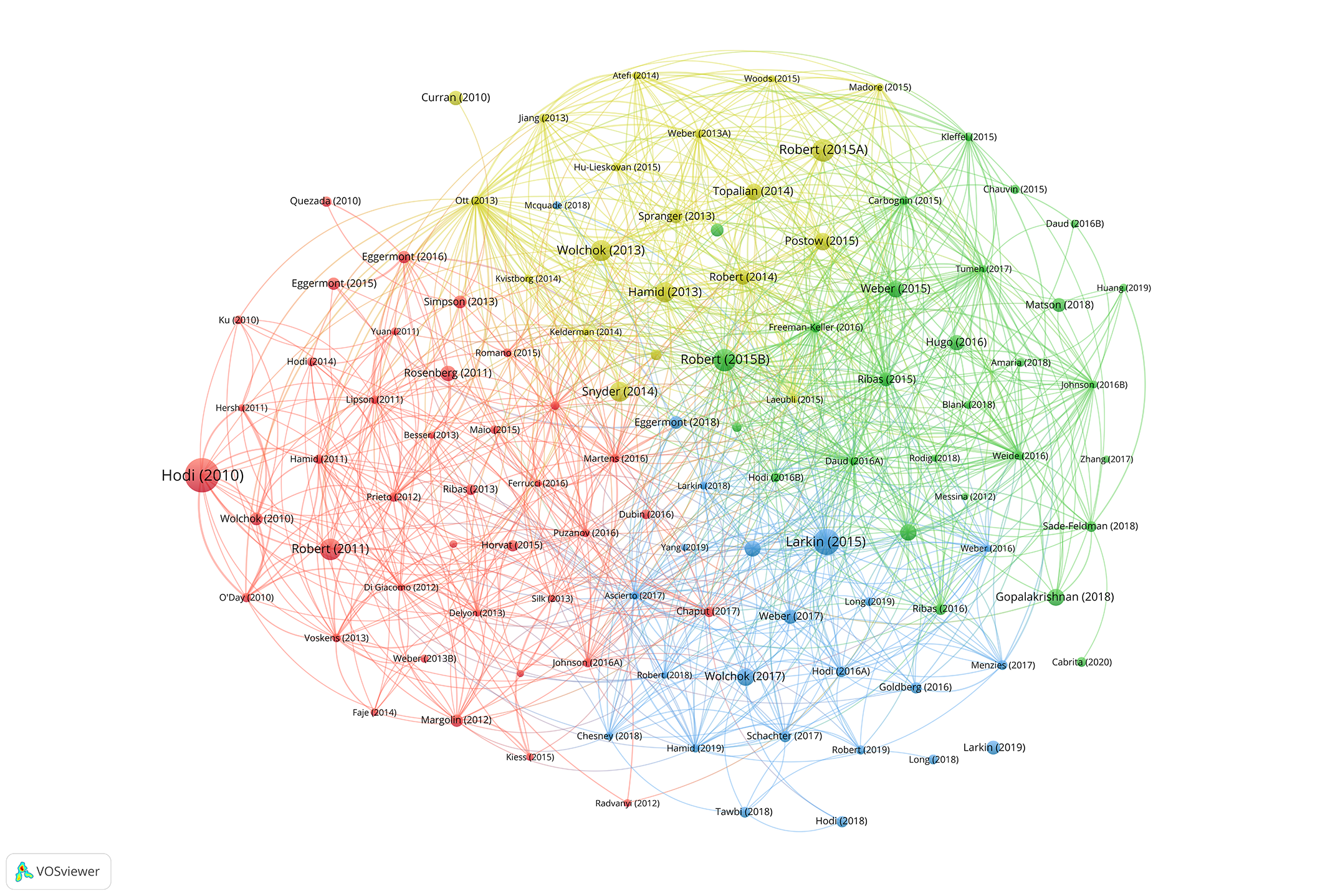

Supplement: Supplementary Figure 3 — Visualization of citation relationship between the papers with the strongest link strength. [file Image_3.png]

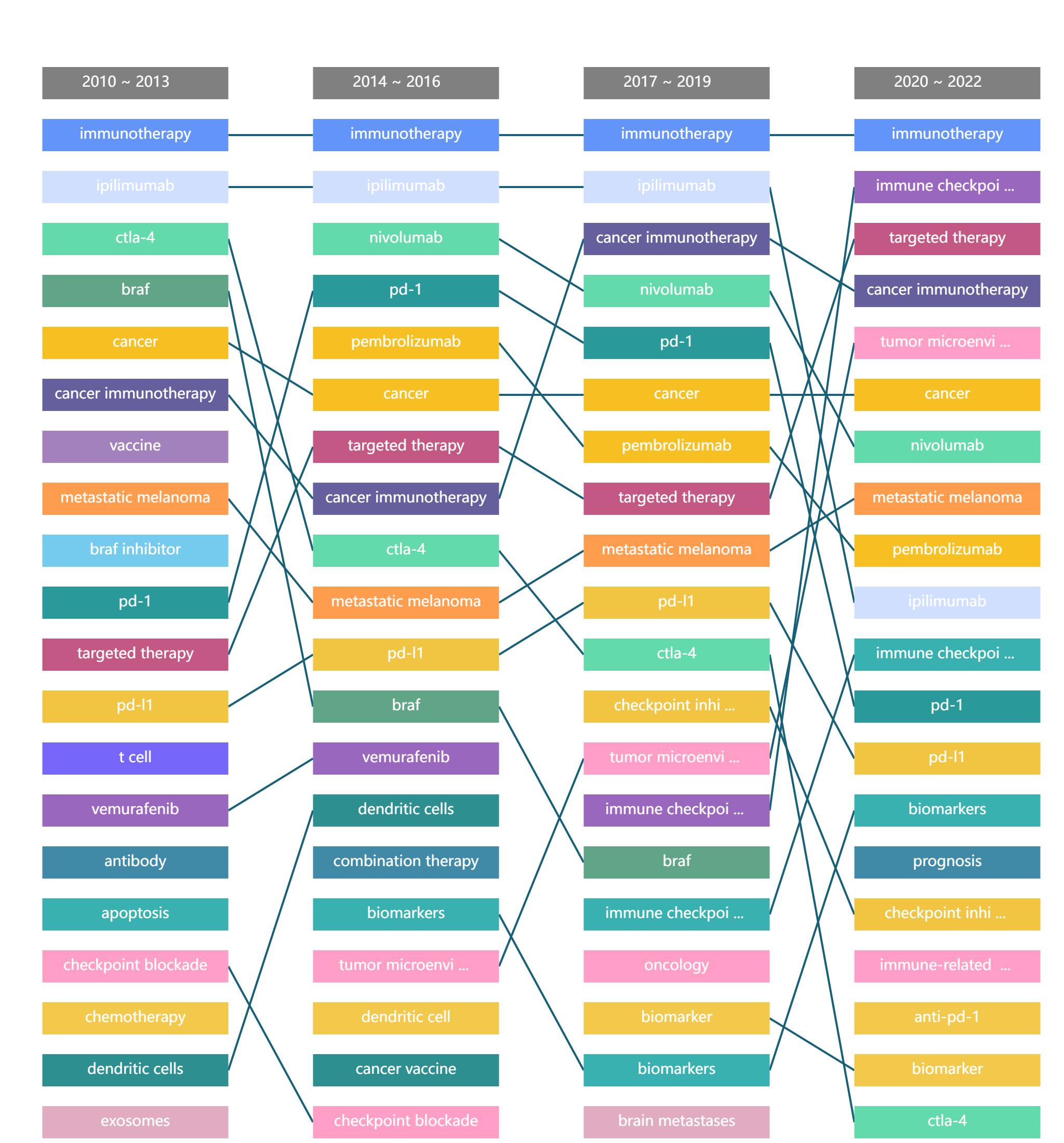

Supplement: Supplementary Figure 4 — The trends of the keywords rank of melanoma immunotherapy from 2010 to 2022. [file Image_4.png]
