# Supplementary material for: Research landscape and trends of melanoma immunotherapy: A bibliometric analysis
Source: Front Oncol. 2023 Jan 9;12:1024179. doi: 10.3389/fonc.2022.1024179 (PMC9868470; doi:10.3389/fonc.2022.1024179)
Supplement: Supplementary Material S1 — Detailed search strategy for the papers on melanoma immunotherapy. [file DataSheet_1.docx]

**Search Strategy**

(TI= (melanoma) AND TI=((immunotherapy) OR (checkpoint inhibitor) OR (checkpoint inhibitors) OR (checkpoint blockade) OR (PD1) OR (PD-1) OR (PD-L1) OR (anti-PD1) OR (anti-PD-1) OR (anti-PD-L1) OR (PD1/PD-L1) OR (PD-1/PD-L1) OR (anti-PD1/PD-L1) OR (anti-PD-1/PD-L1) OR (atezolizumab) OR (avelumab) OR (durvalumab) OR (nivolumab) OR (pembrolizumab) OR (tislelizumab) OR (camrelizumab) OR (penpulimab) OR (toripalimab) OR (sintilimab) OR (lambrolizumab) OR (pidilizumab) OR (cemiplimab) OR (CTL-associated antigen-4) OR (cytotoxic T lymphocyte associated antigen 4) OR (CTLA-4) OR (ipilimumab) OR (tremelimumab) OR (T-Cell Transfer) OR (adoptive cell therapy) OR (CAR-T) OR (chimeric antigen receptor T-cell immunotherapy) OR (talimogene laherparepvec) OR (T-VEC))) AND DT=(Article) NOT TI=((guideline) OR (consensus recommendations) OR (meta-analyses) OR (meta-analysis) OR (meta analysis) OR (data pooling) OR (pooled) OR (overview) OR (current status) OR (development) OR (review) OR (progress to date))
