# Supplementary material for: Research landscape and trends of melanoma immunotherapy: A bibliometric analysis
Source: Front Oncol. 2023 Jan 9;12:1024179. doi: 10.3389/fonc.2022.1024179 (PMC9868470; doi:10.3389/fonc.2022.1024179)
Supplement: Supplementary Table 1 — The 100 most cited papers on melanoma immunotherapy. [file Table_1.docx]

|  | **TABLE S1** The 100 most cited papers in melanoma immunotherapy. | | | | | | | |
| --- | --- | --- | --- | --- | --- | --- | --- | --- |
| Rank | | Title | Corresponding author | Journal | Year | Total citation | Average citation per year (rank) |  |
| 1 | | Improved Survival with Ipilimumab in Patients with Metastatic Melanoma | Hodi FS | N. Engl. J. Med. | 2010 | 9824 | 818.7 (1) |  |
| 2 | | Combined Nivolumab and Ipilimumab or Monotherapy in Untreated Melanoma | Hodi FS | N. Engl. J. Med. | 2015 | 4953 | 707.6 (2) |  |
| 3 | | Nivolumab in Previously Untreated Melanoma without BRAF Mutation | Robert C | N. Engl. J. Med. | 2015 | 3549 | 507 (3) |  |
| 4 | | Pembrolizumab versus Ipilimumab in Advanced Melanoma | Robert C | N. Engl. J. Med. | 2015 | 3506 | 500.9 (4) |  |
| 5 | | Ipilimumab plus Dacarbazine for Previously Untreated Metastatic Melanoma | Wolchok JD | N. Engl. J. Med. | 2011 | 3113 | 283 (10) |  |
| 6 | | Nivolumab plus Ipilimumab in Advanced Melanoma | Wolchok JD | N. Engl. J. Med. | 2013 | 2940 | 326.7 (9) |  |
| 7 | | Genetic Basis for Clinical Response to CTLA-4 Blockade in Melanoma | Chan TA | N. Engl. J. Med. | 2014 | 2614 | 326.8 (8) |  |
| 8 | | Safety and Tumor Responses with Lambrolizumab (Anti-PD-1) in Melanoma | Ribas A | N. Engl. J. Med. | 2013 | 2496 | 277.3 (11) |  |
| 9 | | Nivolumab and Ipilimumab versus Ipilimumab in Untreated Melanoma | Hodi FS | N. Engl. J. Med. | 2015 | 1856 | 265.1 (14) |  |
| 10 | | Overall Survival with Combined Nivolumab and Ipilimumab in Advanced Melanoma | Wolchok JD | N. Engl. J. Med. | 2017 | 1840 | 368 (6) |  |
| 11 | | Nivolumab versus chemotherapy in patients with advanced melanoma who progressed after anti-CTLA-4 treatment (CheckMate 037): a randomised, controlled, open-label, phase 3 trial | Weber JS | Lancet Oncol. | 2015 | 1761 | 251.6 (15) |  |
| 12 | | Gut microbiome modulates response to anti-PD-1 immunotherapy in melanoma patients | Wargo JA | Science | 2018 | 1692 | 423 (5) |  |
| 13 | | Survival, Durable Tumor Remission, and Long-Term Safety in Patients With Advanced Melanoma Receiving Nivolumab | Topalian SL | J. Clin. Oncol. | 2014 | 1611 | 201.4 (21) |  |
| 14 | | Mutations Associated with Acquired Resistance to PD-1 Blockade in Melanoma | Ribas A | N. Engl. J. Med. | 2016 | 1598 | 266.3 (13) |  |
| 15 | | Genomic correlates of response to CTLA-4 blockade in metastatic melanoma | Garraway LA | Science | 2015 | 1437 | 205.3 (20) |  |
| 16 | | Genomic and Transcriptomic Features of Response to Anti-PD-1 Therapy in Metastatic Melanoma | Lo RS | Cell | 2016 | 1390 | 231.7 (16) |  |
| 17 | | Durable Complete Responses in Heavily Pretreated Patients with Metastatic Melanoma Using T-Cell Transfer Immunotherapy | Rosenberg SA | Clin. Cancer Res. | 2011 | 1332 | 121.1 (32) |  |
| 18 | | Anti-programmed-death-receptor-1 treatment with pembrolizumab in ipilimumab-refractory advanced melanoma: a randomised dose-comparison cohort of a phase 1 trial | Robert C | Lancet | 2014 | 1279 | 159.9 (23) |  |
| 19 | | PD-1 and CTLA-4 combination blockade expands infiltrating T cells and reduces regulatory T and myeloid cells within B16 melanoma tumors | Allison JP | Proc. Natl. Acad. Sci. U. S. A. | 2010 | 1175 | 97.9 (40) |  |
| 20 | | Adjuvant Nivolumab versus Ipilimumab in Resected Stage III or IV Melanoma | Weber J | N. Engl. J. Med. | 2017 | 1102 | 220.4 (18) |  |
| 21 | | Up-Regulation of PD-L1, IDO, and T-regs in the Melanoma Tumor Microenvironment Is Driven by CD8(+) T Cells | Spranger S | Sci. Transl. Med. | 2013 | 1077 | 119.7 (33) |  |
| 22 | | The commensal microbiome is associated with anti-PD-1 efficacy in metastatic melanoma patients | Gajewski TF | Science | 2018 | 1076 | 269 (12) |  |
| 23 | | Five-Year Survival with Combined Nivolumab and Ipilimumab in Advanced Melanoma | Larkin J | N. Engl. J. Med. | 2019 | 1074 | 358 (7) |  |
| 24 | | Pembrolizumab versus investigator-choice chemotherapy for ipilimumab-refractory melanoma (KEYNOTE-002): a randomised, controlled, phase 2 trial | Ribas A | Lancet Oncol. | 2015 | 1058 | 151.1 (25) |  |
| 25 | | Fc-dependent depletion of tumor-infiltrating regulatory T cells co-defines the efficacy of anti-CTLA-4 therapy against melanoma | Quezada SA | J. Exp. Med. | 2013 | 884 | 98.2 (39) |  |
| 26 | | Upregulation of Tim-3 and PD-1 expression is associated with tumor antigen-specific CD8(+) T cell dysfunction in melanoma patients | Zarour HM | J. Exp. Med. | 2010 | 849 | 70.8 (57) |  |
| 27 | | Adjuvant Pembrolizumab versus Placebo in Resected Stage III Melanoma | Eggermont AMM | N. Engl. J. Med. | 2018 | 839 | 209.8 (19) |  |
| 28 | | Ipilimumab monotherapy in patients with pretreated advanced melanoma: a randomised, double-blind, multicentre, phase 2, dose-ranging study | Wolchok JD | Lancet Oncol. | 2010 | 839 | 69.9 (58) |  |
| 29 | | Prolonged Survival in Stage III Melanoma with Ipilimumab Adjuvant Therapy | Eggermont AMM | N. Engl. J. Med. | 2016 | 809 | 134.8 (29) |  |
| 30 | | Adjuvant ipilimumab versus placebo after complete resection of high-risk stage III melanoma (EORTC 18071): a randomised, double-blind, phase 3 trial | Eggermont AMM | Lancet Oncol. | 2015 | 781 | 111.6 (35) |  |
| 31 | | Ipilimumab in patients with melanoma and brain metastases: an open-label, phase 2 trial | Margolin K | Lancet Oncol. | 2012 | 746 | 74.6 (53) |  |
| 32 | | Pembrolizumab versus ipilimumab for advanced melanoma: final overall survival results of a multicentre, randomised, open-label phase 3 study (KEYNOTE-006) | Schachter J | Lancet | 2017 | 656 | 131.2 (30) |  |
| 33 | | Association of Pembrolizumab With Tumor Response and Survival Among Patients With Advanced Melanoma | Ribas A | JAMA-J. Am. Med. Assoc. | 2016 | 643 | 107.2 (37) |  |
| 34 | | Nivolumab plus ipilimumab or nivolumab alone versus ipilimumab alone in advanced melanoma (CheckMate 067): 4-year outcomes of a multicentre, randomised, phase 3 trial | Hodi FS | Lancet Oncol. | 2018 | 627 | 156.8 (24) |  |
| 35 | | Pembrolizumab for patients with melanoma or non-small-cell lung cancer and untreated brain metastases: early analysis of a non-randomised, open-label, phase 2 trial | Goldberg SB | Lancet Oncol. | 2016 | 616 | 102.7 (38) |  |
| 36 | | Immune-Related Adverse Events, Need for Systemic Immunosuppression, and Effects on Survival and Time to Treatment Failure in Patients With Melanoma Treated With Ipilimumab at Memorial Sloan Kettering Cancer Center | Chapman PB | J. Clin. Oncol. | 2015 | 614 | 87.7 (45) |  |
| 37 | | Tumor Exome Analysis Reveals Neoantigen-Specific T-Cell Reactivity in an Ipilimumab-Responsive Melanoma | van Rooij N | J. Clin. Oncol. | 2013 | 584 | 64.9 (62) |  |
| 38 | | Defining T Cell States Associated with Response to Checkpoint Immunotherapy in Melanoma | Getz G; Hacohen N | Cell | 2018 | 565 | 141.3 (26) |  |
| 39 | | Combined nivolumab and ipilimumab versus ipilimumab alone in patients with advanced melanoma: 2-year overall survival outcomes in a multicentre, randomised, controlled, phase 2 trial | Hodi FS | Lancet Oncol. | 2016 | 565 | 94.2 (43) |  |
| 40 | | Combined Nivolumab and Ipilimumab in Melanoma Metastatic to the Brain | Tawbi HA | N. Engl. J. Med. | 2018 | 563 | 140.8 (27) |  |
| 41 | | Phase III Randomized Clinical Trial Comparing Tremelimumab With Standard-of-Care Chemotherapy in Patients With Advanced Melanoma | Ribas A | J. Clin. Oncol. | 2013 | 550 | 61.1 (65) |  |
| 42 | | Tumor-reactive CD4(+) T cells develop cytotoxic activity and eradicate large established melanoma after transfer into lymphopenic hosts | Allison JP | J. Exp. Med. | 2010 | 529 | 44.1 (94) |  |
| 43 | | Baseline gut microbiota predicts clinical response and colitis in metastatic melanoma patients treated with ipilimumab | Carbonnel F | Ann. Oncol. | 2017 | 482 | 96.4 (41) |  |
| 44 | | Nivolumab in Resected and Unresectable Metastatic Melanoma: Characteristics of Immune-Related Adverse Events and Association with Outcomes | Freeman-Keller M | Clin. Cancer Res. | 2016 | 480 | 80 (48) |  |
| 45 | | Intestinal microbiome analyses identify melanoma patients at risk for checkpoint-blockade-induced colitis | Pamer EG | Nat. Commun. | 2016 | 465 | 77.5 (50) |  |
| 46 | | Evaluation of Immune-Related Response Criteria and RECIST v1.1 in Patients With Advanced Melanoma Treated With Pembrolizumab | Hodi FS | J. Clin. Oncol. | 2016 | 462 | 77 (51) |  |
| 47 | | Tertiary lymphoid structures improve immunotherapy and survival in melanoma | Jonsson G | Nature | 2020 | 453 | 226.5 (17) |  |
| 48 | | CTLA-4 and PD-1/PD-L1 Blockade: New Immunotherapeutic Modalities with Durable Clinical Benefit in Melanoma Patients | Robert C | Clin. Cancer Res. | 2013 | 452 | 50.2 (81) |  |
| 49 | | Anti-PD-1 therapy in patients with advanced melanoma and preexisting autoimmune disorders or major toxicity with ipilimumab | Menzies AM | Ann. Oncol. | 2017 | 450 | 90 (44) |  |
| 50 | | Combination nivolumab and ipilimumab or nivolumab alone in melanoma brain metastases: a multicentre randomised phase 2 study | Long GV | Lancet Oncol. | 2018 | 435 | 108.8 (36) |  |
| 51 | | Safety, Efficacy, and Biomarkers of Nivolumab With Vaccine in Ipilimumab-Refractory or -Naive Melanoma | Weber JS | J. Clin. Oncol. | 2013 | 402 | 44.7 (92) |  |
| 52 | | Ipilimumab Therapy in Patients With Advanced Melanoma and Preexisting Autoimmune Disorders | Johnson DB | JAMA Oncol. | 2016 | 396 | 66 (60) |  |
| 53 | | Pembrolizumab versus ipilimumab in advanced melanoma (KEYNOTE-006): post-hoc 5-year results from an open-label, multicentre, randomised, controlled, phase 3 study | Robert C | Lancet Oncol. | 2019 | 393 | 131 (31) |  |
| 54 | | A prospective phase II trial exploring the association between tumor microenvironment biomarkers and clinical activity of ipilimumab in advanced melanoma | Hamid O | J. Transl. Med. | 2011 | 390 | 35.5 (122) |  |
| 55 | | Bevacizumab plus Ipilimumab in Patients with Metastatic Melanoma | Hodi FS | Cancer Immunol. Res. | 2014 | 389 | 48.6 (85) |  |
| 56 | | TIGIT and PD-1 impair tumor antigen-specific CD8(+) T cells in melanoma patients | Zarour HM | J. Clin. Invest. | 2015 | 385 | 55 (75) |  |
| 57 | | Efficacy and safety of ipilimumab monotherapy in patients with pretreated advanced melanoma: a multicenter single-arm phase II study | O'Day SJ | Ann. Oncol. | 2010 | 384 | 32 (135) |  |
| 58 | | Association of Vitiligo With Tumor Response in Patients With Metastatic Melanoma Treated With Pembrolizumab | Robert C | JAMA Dermatol. | 2016 | 375 | 62.5 (64) |  |
| 59 | | CTLA-4 Blockade with Ipilimumab: Long-term Follow-up of 177 Patients with Metastatic Melanoma | Phan GQ | Clin. Cancer Res. | 2012 | 362 | 36.2 (118) |  |
| 60 | | Ipilimumab-dependent cell-mediated cytotoxicity of regulatory T cells ex vivo by nonclassical monocytes in melanoma patients | Romano E | Proc. Natl. Acad. Sci. U. S. A. | 2015 | 355 | 50.7 (79) |  |
| 61 | | Programmed Death-Ligand 1 Expression and Response to the Anti-Programmed Death 1 Antibody Pembrolizumab in Melanoma | Daud AI | J. Clin. Oncol. | 2016 | 352 | 58.7 (67) |  |
| 62 | | Improved antitumor activity of immunotherapy with BRAF and MEK inhibitors in BRAF(V600E) melanoma | Ribas A | Sci. Transl. Med. | 2015 | 346 | 49.4 (83) |  |
| 63 | | Single-Institution Experience With Ipilimumab in Advanced Melanoma Patients in the Compassionate Use Setting Lymphocyte Count After 2 Doses Correlates With Survival | Wolchok JD | Cancer | 2010 | 341 | 28.4 (152) |  |
| 64 | | Melanoma Cell-Intrinsic PD-1 Receptor Functions Promote Tumor Growth | Schatton T | Cell | 2015 | 338 | 48.3 (86) |  |
| 65 | | Baseline Peripheral Blood Biomarkers Associated with Clinical Outcome of Advanced Melanoma Patients Treated with Ipilimumab | Weide B | Clin. Cancer Res. | 2016 | 337 | 56.2 (70) |  |
| 66 | | Epacadostat plus pembrolizumab versus placebo plus pembrolizumab in patients with unresectable or metastatic melanoma (ECHO-301/KEYNOTE-252): a phase 3, randomised, double-blind study | Long GV | Lancet Oncol. | 2019 | 336 | 112 (34) |  |
| 67 | | Baseline Biomarkers for Outcome of Melanoma Patients Treated with Pembrolizumab | Weide B | Clin. Cancer Res. | 2016 | 336 | 56 (71) |  |
| 68 | | Differential Activity of Nivolumab, Pembrolizumab and MPDL3280A according to the Tumor Expression of Programmed Death-Ligand-1 (PD-L1): Sensitivity Analysis of Trials in Melanoma, Lung and Genitourinary Cancers | Bria E | PLoS One | 2015 | 333 | 47.6 (87) |  |
| 69 | | Neoadjuvant immune checkpoint blockade in high-risk resectable melanoma | Wargo JA | Nat. Med. | 2018 | 330 | 82.5 (46) |  |
| 70 | | Frequencies of circulating MDSC correlate with clinical outcome of melanoma patients treated with ipilimumab | Speiser DE | Cancer Immunol. Immunother. | 2014 | 321 | 40.1 (103) |  |
| 71 | | The Activation of MAPK in Melanoma Cells Resistant to BRAF Inhibition Promotes PD-L1 Expression That Is Reversible by MEK and PI3K Inhibition | Hodi FS | Clin. Cancer Res. | 2013 | 321 | 35.7 (121) |  |
| 72 | | The Price of Tumor Control: An Analysis of Rare Side Effects of Anti-CTLA-4 Therapy in Metastatic Melanoma from the Ipilimumab Network | Heinzerling LM | PLoS One | 2013 | 318 | 35.3 (123) |  |
| 73 | | Five-Year Survival Rates for Treatment-Naive Patients With Advanced Melanoma Who Received Ipilimumab Plus Dacarbazine in a Phase III Trial | Maio M | J. Clin. Oncol. | 2015 | 314 | 44.9 (91) |  |
| 74 | | Talimogene Laherparepvec in Combination With Ipilimumab in Previously Untreated, Unresectable Stage IIIB-IV Melanoma | Puzanov I | J. Clin. Oncol. | 2016 | 310 | 51.7 (77) |  |
| 75 | | Randomized, Open-Label Phase II Study Evaluating the Efficacy and Safety of Talimogene Laherparepvec in Combination With Ipilimumab Versus Ipilimumab Alone in Patients With Advanced, Unresectable Melanoma | Chesney J | J. Clin. Oncol. | 2018 | 305 | 76.3 (52) |  |
| 76 | | Tumor immune profiling predicts response to anti-PD-1 therapy in human melanoma | Rosenblum MD | J. Clin. Invest. | 2016 | 303 | 50.5 (80) |  |
| 77 | | Neoadjuvant versus adjuvant ipilimumab plus nivolumab in macroscopic stage III melanoma | Blank CU | Nat. Med. | 2018 | 298 | 74.5 (54) |  |
| 78 | | Patterns of Onset and Resolution of Immune-Related Adverse Events of Special Interest With Ipilimumab Detailed Safety Analysis From a Phase 3 Trial in Patients With Advanced Melanoma | Weber JS | Cancer | 2013 | 296 | 32.9 (131) |  |
| 79 | | Five-year survival outcomes for patients with advanced melanoma treated with pembrolizumab in KEYNOTE-001 | Hamid O | Ann. Oncol. | 2019 | 284 | 94.7 (42) |  |
| 80 | | Ipilimumab: An Anti-CTLA-4 Antibody for Metastatic Melanoma | Drake CG | Clin. Cancer Res. | 2011 | 284 | 25.8 (165) |  |
| 81 | | PD-L1 expression in melanoma shows marked heterogeneity within and between patients: implications for anti-PD-1/PD-L1 clinical trials | Scolyer RA | Pigment Cell Melanoma Res. | 2015 | 282 | 40.3 (102) |  |
| 82 | | Ipilimumab 10 mg/kg versus ipilimumab 3 mg/kg in patients with unresectable or metastatic melanoma: a randomised, double-blind, multicentre, phase 3 trial | Ascierto PA | Lancet Oncol. | 2017 | 277 | 55.4 (74) |  |
| 83 | | Association of body-mass index and outcomes in patients with metastatic melanoma treated with targeted therapy, immunotherapy, or chemotherapy: a retrospective, multicohort analysis | McQuade JL | Lancet Oncol. | 2018 | 274 | 68.5 (59) |  |
| 84 | | Specific Lymphocyte Subsets Predict Response to Adoptive Cell Therapy Using Expanded Autologous Tumor-Infiltrating Lymphocytes in Metastatic Melanoma Patients | Radvanyi LG | Clin. Cancer Res. | 2012 | 262 | 26.2 (162) |  |
| 85 | | Anti-CTLA-4 therapy broadens the melanoma-reactive CD8(+) T cell response | Kvistborg P | Sci. Transl. Med. | 2014 | 261 | 32.6 (132) |  |
| 86 | | Stereotactic Radiosurgery for Melanoma Brain Metastases in Patients Receiving Ipilimumab: Safety Profile and Efficacy of Combined Treatment | Beal K | Int. J. Radiat. Oncol. Biol. Phys. | 2015 | 255 | 36.4 (117) |  |
| 87 | | Ipilimumab-Induced Hypophysitis: A Detailed Longitudinal Analysis in a Large Cohort of Patients With Metastatic Melanoma | Faje AT | J. Clin. Endocrinol. Metab. | 2014 | 249 | 31.1 (141) |  |
| 88 | | Melanoma-specific MHC-II expression represents a tumour-autonomous phenotype and predicts response to anti-PD-1/PD-L1 therapy | Johnson DB; Balko JM | Nat. Commun. | 2016 | 247 | 41.2 (101) |  |
| 89 | | Integrated NY-ESO-1 antibody and CD8(+) T-cell responses correlate with clinical benefit in advanced melanoma patients treated with ipilimumab | Old LJ | Proc. Natl. Acad. Sci. U. S. A. | 2011 | 245 | 22.3 (197) |  |
| 90 | | A single dose of neoadjuvant PD-1 blockade predicts clinical outcomes in resectable melanoma | Huang AC; Mitchell TC | Nat. Med. | 2019 | 242 | 80.7 (47) |  |
| 91 | | Overall Survival in Patients With Advanced Melanoma Who Received Nivolumab Versus Investigator's Choice Chemotherapy in CheckMate 037: A Randomized, Controlled, Open-Label Phase III Trial | Larkin J | J. Clin. Oncol. | 2018 | 240 | 60 (66) |  |
| 92 | | Radiosurgery for melanoma brain metastases in the ipilimumab era and the possibility of longer survival Clinical article | Knisely JPS | J. Neurosurg. | 2012 | 240 | 24 (178) |  |
| 93 | | Effects of MAPK and PI3K Pathways on PD-L1 Expression in Melanoma | Ribas A | Clin. Cancer Res. | 2014 | 237 | 29.6 (147) |  |
| 94 | | Baseline neutrophils and derived neutrophil-to-lymphocyte ratio: prognostic relevance in metastatic melanoma patients receiving ipilimumab | Martinoli C | Ann. Oncol. | 2016 | 228 | 38 (109) |  |
| 95 | | Experience in daily practice with ipilimumab for the treatment of patients with metastatic melanoma: an early increase in lymphocyte and eosinophil counts is associated with improved survival | Delyon J | Ann. Oncol. | 2013 | 227 | 25.2 (167) |  |
| 96 | | 12-Chemokine Gene Signature Identifies Lymph Node-like Structures in Melanoma: Potential for Patient Selection for Immunotherapy? | Mule JJ | Sci Rep | 2012 | 224 | 22.4 (195) |  |
| 97 | | Enhancing CD8(+) T Cell Fatty Acid Catabolism within a Metabolically Challenging Tumor Microenvironment Increases the Efficacy of Melanoma Immunotherapy | Ertl HCJ | Cancer Cell | 2017 | 222 | 44.4 (93) |  |
| 98 | | Sequential administration of nivolumab and ipilimumab with a planned switch in patients with advanced melanoma (CheckMate 064): an open-label, randomised, phase 2 trial | Hodi FS | Lancet Oncol. | 2016 | 220 | 36.7 (115) |  |
| 99 | | Liver Metastasis and Treatment Outcome with Anti-PD-1 Monoclonal Antibody in Patients with Melanoma and NSCLC | Daud A | Cancer Immunol. Res. | 2017 | 218 | 43.6 (96) |  |
| 100 | | HDAC Inhibition Upregulates PD-1 Ligands in Melanoma and Augments Immunotherapy with PD-1 Blockade | Weber J | Cancer Immunol. Res. | 2015 | 218 | 31.1 (140) |  |
