# Supplementary material for: Research landscape and trends of melanoma immunotherapy: A bibliometric analysis
Source: Front Oncol. 2023 Jan 9;12:1024179. doi: 10.3389/fonc.2022.1024179 (PMC9868470; doi:10.3389/fonc.2022.1024179)
Supplement: Supplementary Table 2 — The journals with top-cited papers on melanoma immunotherapy. [file Table_2.docx]

| **TABLE S2** \| The journals with top-cited papers in melanoma immunotherapy from 2010 to 2022. | | | | |
| --- | --- | --- | --- | --- |
| **Journals with top-cited papers** | **Top-Cited Paper number** | **Paper number** | **Paper number (2010-2020)** | **TPR (2010-2020)** |
| N. Engl. J. Med. | 16 | 17 | 16 | 100.0% |
| Lancet Oncol. | 14 | 25 | 20 | 70.0% |
| J. Clin. Oncol. | 11 | 40 | 35 | 31.4% |
| Clin. Cancer Res. | 10 | 80 | 68 | 14.7% |
| Ann. Oncol. | 6 | 38 | 32 | 18.8% |
| Science | 3 | 6 | 3 | 100.0% |
| Cell | 3 | 5 | 5 | 60.0% |
| J. Exp. Med. | 3 | 3 | 3 | 100.0% |
| Sci. Transl. Med. | 3 | 7 | 7 | 42.9% |
| Cancer Immunol. Res. | 3 | 47 | 43 | 7.0% |
| Nat. Med. | 3 | 19 | 13 | 23.1% |
| Proc. Natl. Acad. Sci. U. S. A. | 3 | 10 | 9 | 33.3% |
| Lancet | 2 | 4 | 4 | 50.0% |
| Cancer | 2 | 16 | 14 | 14.3% |
| J. Clin. Invest. | 2 | 7 | 5 | 40.0% |
| Nat. Commun. | 2 | 15 | 13 | 15.4% |
| PLoS One | 2 | 28 | 26 | 7.7% |
| Nature | 1 | 3 | 3 | 33.3% |
| JAMA | 1 | 2 | 2 | 50.0% |
| JAMA Oncol. | 1 | 8 | 6 | 16.7% |
| Cancer Cell | 1 | 3 | 2 | 50.0% |
| Cancer Immunol. Immunother. | 1 | 71 | 57 | 1.8% |
| Int. J. Radiat. Oncol. Biol. Phys. | 1 | 12 | 11 | 9.1% |
| JAMA Dermatol. | 1 | 7 | 6 | 16.7% |
| J. Clin. Endocrinol. Metab. | 1 | 2 | 2 | 50.0% |
| J. Neurosurg. | 1 | 3 | 3 | 33.3% |
| J. Transl. Med. | 1 | 22 | 22 | 4.5% |
| Pigment Cell Melanoma Res. | 1 | 17 | 12 | 8.3% |
| Sci Rep | 1 | 12 | 9 | 11.1% |
|  | | | | |
