# Supplementary material for: Research landscape and trends of melanoma immunotherapy: A bibliometric analysis
Source: Front Oncol. 2023 Jan 9;12:1024179. doi: 10.3389/fonc.2022.1024179 (PMC9868470; doi:10.3389/fonc.2022.1024179)
Supplement: Supplementary Table 3 — The papers on melanoma immunotherapy published in major journals since 2020. [file Table_3.docx]

|  | **TABLE S3** The 118 papers in melanoma immunotherapy published in major journals since 2020. | | | | | |
| --- | --- | --- | --- | --- | --- | --- |
| No. | | Title | Corresponding author | Journal | Year | Total citation |
| 1 | | A Phase Ib Trial of Personalized Neoantigen Therapy Plus Anti-PD-1 in Patients with Advanced Melanoma, Non-small Cell Lung Cancer, or Bladder Cancer | Ott PA | Cell | 2020 | 106 |
| 2 | | A Prospective, Phase 1 Trial of Nivolumab, Ipilimumab, and Radiotherapy in Patients with Advanced Melanoma | Postow MA | Clin. Cancer Res. | 2020 | 13 |
| 3 | | Adjuvant nivolumab plus ipilimumab or nivolumab monotherapy versus placebo in patients with resected stage IV melanoma with no evidence of disease (IMMUNED): a randomised, double-blind, placebo-controlled, phase 2 trial | Schadendorf D | Lancet | 2020 | 78 |
| 4 | | Adjuvant nivolumab versus ipilimumab in resected stage IIIB-C and stage IV melanoma (CheckMate 238): 4-year results from a multicentre, double-blind, randomised, controlled, phase 3 trial | Ascierto PA | Lancet Oncol. | 2020 | 121 |
| 5 | | An RNA vaccine drives immunity in checkpoint-inhibitor-treated melanoma | Sahin U | Nature | 2020 | 159 |
| 6 | | Anti-PD1 checkpoint inhibitor therapy in acral melanoma: a multicenter study of 193 Japanese patients | Nakamura Y | Ann. Oncol. | 2020 | 28 |
| 7 | | Assessment of clinical outcomes with immune checkpoint inhibitor therapy in melanoma patients with CDKN2A and TP53 pathogenic mutations | Bryce AH | PLoS One | 2020 | 8 |
| 8 | | Association Between Immune-Related Adverse Events and Recurrence-Free Survival Among Patients With Stage III Melanoma Randomized to Receive Pembrolizumab or Placebo A Secondary Analysis of a Randomized Clinical Trial | Suciu S | JAMA Oncol. | 2020 | 133 |
| 9 | | Association of Anti-Programmed Cell Death 1 Antibody Treatment With Risk of Recurrence of Toxic Effects After Immune-Related Adverse Events of Ipilimumab in Patients With Metastatic Melanoma | Brunot A | JAMA Dermatol. | 2020 | 6 |
| 10 | | Association of Anti-TNF with Decreased Survival in Steroid Refractory Ipilimumab and Anti-PD1-Treated Patients in the Dutch Melanoma Treatment Registry | Suijkerbuijk KPM | Clin. Cancer Res. | 2020 | 53 |
| 11 | | Atezolizumab, vemurafenib, and cobimetinib as first-line treatment for unresectable advanced BRAF(V600) mutation-positive melanoma (IMspire150): primary analysis of the randomised, double-blind, placebo-controlled, phase 3 trial | Gutzmer R | Lancet | 2020 | 167 |
| 12 | | Baseline IFN-gamma and IL-10 expression in PBMCs could predict response to PD-1 checkpoint inhibitors in advanced melanoma patients | Troiani T | Sci Rep | 2020 | 5 |
| 13 | | Checkpoint inhibitors: Better outcomes among advanced cutaneous head and neck melanoma patients | Hirshoren N | PLoS One | 2020 | 2 |
| 14 | | Circulating Tumor DNA Predicts Outcome from First-, but not Second-line Treatment and Identifies Melanoma Patients Who May Benefit from Combination Immunotherapy | Gray ES | Clin. Cancer Res. | 2020 | 17 |
| 15 | | Combined ipilimumab and nivolumab first-line and after BRAF-targeted therapy in advanced melanoma | Mason R | Pigment Cell Melanoma Res. | 2020 | 17 |
| 16 | | Combined PD-1, BRAF and MEK inhibition in advanced BRAF-mutant melanoma: safety run-in and biomarker cohorts of COMBI-i | Dummer R | Nat. Med. | 2020 | 31 |
| 17 | | Conserved Interferon-gamma Signaling Drives Clinical Response to Immune Checkpoint Blockade Therapy in Melanoma | Grasso CS; Ribas A | Cancer Cell | 2020 | 72 |
| 18 | | Elevated baseline serum PD-1 or PD-L1 predicts poor outcome of PD-1 inhibition therapy in metastatic melanoma | Ugurel S | Ann. Oncol. | 2020 | 31 |
| 19 | | Five-Year Outcomes With Nivolumab in Patients With Wild-Type BRAF Advanced Melanoma | Robert C | J. Clin. Oncol. | 2020 | 37 |
| 20 | | Frontline BRAF Testing-Guided Treatment for Advanced Melanoma in the Era of Immunotherapies: A Cost-Utility Analysis Based on Long-term Survival Data | Shi LZ | JAMA Dermatol. | 2020 | 5 |
| 21 | | High neutrophil-to-lymphocyte ratio (NLR) is associated with treatment failure and death in patients who have melanoma treated with PD-1 inhibitor monotherapy | Bartlett EK | Cancer | 2020 | 44 |
| 22 | | Identification of Small Molecule Enhancers of Immunotherapy for Melanoma | Ferrer M; Singh A | Sci Rep | 2020 | 3 |
| 23 | | Impact of Sequencing Radiation Therapy and Immune Checkpoint Inhibitors in the Treatment of Melanoma Brain Metastases | Sengupta S | Int. J. Radiat. Oncol. Biol. Phys. | 2020 | 10 |
| 24 | | Longer Follow-Up Confirms Recurrence-Free Survival Benefit of Adjuvant Pembrolizumab in High-Risk Stage III Melanoma: Updated Results From the EORTC 1325-MG/KEYNOTE-054 Trial | Eggermont AMM | J. Clin. Oncol. | 2020 | 66 |
| 25 | | Longitudinal Monitoring of ctDNA in Patients with Melanoma and Brain Metastases Treated with Immune Checkpoint Inhibitors | Lee JH | Clin. Cancer Res. | 2020 | 23 |
| 26 | | Long-term Follow-up of Standard-Dose Pembrolizumab Plus Reduced-Dose Ipilimumab in Patients with Advanced Melanoma: KEYNOTE-029 Part 1B | Carlino MS | Clin. Cancer Res. | 2020 | 12 |
| 27 | | Long-Term Outcomes and Responses to Retreatment in Patients With Melanoma Treated With PD-1 Blockade | Warner AB | J. Clin. Oncol. | 2020 | 71 |
| 28 | | Management of early melanoma recurrence despite adjuvant anti-PD-1 antibody therapy | Long GV | Ann. Oncol. | 2020 | 21 |
| 29 | | Melanoma Evolves Complete Immunotherapy Resistance through the Acquisition of a Hypermetabolic Phenotype | Curran MA | Cancer Immunol. Res. | 2020 | 10 |
| 30 | | Multimodel preclinical platform predicts clinical response of melanoma to immunotherapy | Lee MP; Day CP; Merlino G | Nat. Med. | 2020 | 25 |
| 31 | | Outcomes after progression of disease with anti-PD-1/PD-L1 therapy for patients with advanced melanoma | Johnson DB | Cancer | 2020 | 7 |
| 32 | | PCC0208025 (BMS202), a small molecule inhibitor of PD-L1, produces an antitumor effect in B16-F10 melanoma-bearing mice | Ye L | PLoS One | 2020 | 12 |
| 33 | | PD-L1 blockade in combination with inhibition of MAPK oncogenic signaling in patients with advanced melanoma | Ribas A | Nat. Commun. | 2020 | 19 |
| 34 | | PD-L1 expression in equine malignant melanoma and functional effects of PD-L1 blockade | Konnai S | PLoS One | 2020 | 2 |
| 35 | | Pembrolizumab in paediatric patients with advanced melanoma or a PD-L1-positive, advanced, relapsed, or refractory solid tumour or lymphoma (KEYNOTE-051): interim analysis of an open-label, single-arm, phase 1-2 trial | Geoerger B | Lancet Oncol. | 2020 | 86 |
| 36 | | Peripheral CD8(+) T cell characteristics associated with durable responses to immune checkpoint blockade in patients with metastatic melanoma | Fairfax BP | Nat. Med. | 2020 | 86 |
| 37 | | Phase II Trial of IL-12 Plasmid Transfection and PD-1 Blockade in Immunologically Quiescent Melanoma | Daud AI | Clin. Cancer Res. | 2020 | 35 |
| 38 | | Phase III Study of Adjuvant Ipilimumab (3 or 10 mg/kg) Versus High-Dose Interferon Alfa-2b for Resected High-Risk Melanoma: North American Intergroup E1609 | Tarhini AA | J. Clin. Oncol. | 2020 | 47 |
| 39 | | Primary tumor characteristics and next-generation sequencing mutations as biomarkers for melanoma immunotherapy response | Farma JM | Pigment Cell Melanoma Res. | 2020 | 1 |
| 40 | | Real-world analyses of therapy discontinuation of checkpoint inhibitors in metastatic melanoma patients | Bernatsky S | Sci Rep | 2020 | 1 |
| 41 | | Resistance of melanoma to immune checkpoint inhibitors is overcome by targeting the sphingosine kinase-1 | Colacios C | Nat. Commun. | 2020 | 48 |
| 42 | | Safety, Efficacy, and Biomarker Analysis of Toripalimab in Previously Treated Advanced Melanoma: Results of the POLARIS-01 Multicenter Phase II Trial | Guo J | Clin. Cancer Res. | 2020 | 35 |
| 43 | | Stereotactic Radiation Therapy Combined With Immunotherapy Against Metastatic Melanoma: Long-Term Results of a Phase 1 Clinical Trial | Ratnayake G | Int. J. Radiat. Oncol. Biol. Phys. | 2020 | 2 |
| 44 | | Stroma remodeling and reduced cell division define durable response to PD-1 blockade in melanoma | Marais R | Nat. Commun. | 2020 | 8 |
| 45 | | Targeting the innate immunoreceptor RIG-I overcomes melanoma-intrinsic resistance to T cell immunotherapy | Paschen A | J. Clin. Invest. | 2020 | 15 |
| 46 | | Tertiary lymphoid structures improve immunotherapy and survival in melanoma | Jonsson G | Nature | 2020 | 465 |
| 47 | | The prognostic significance of VISTA and CD33-positive myeloid cells in cutaneous melanoma and their relationship with PD-1 expression | Chang SE; Lee WJ | Sci Rep | 2020 | 14 |
| 48 | | Transcriptional downregulation of MHC class I and melanoma de- differentiation in resistance to PD-1 inhibition | Rizos H | Nat. Commun. | 2020 | 3 |
| 49 | | Tumor CD155 Expression Is Associated with Resistance to Anti-PD1 Immunotherapy in Metastatic Melanoma | Smyth MJ | Clin. Cancer Res. | 2020 | 24 |
| 50 | | Uncoupling interferon signaling and antigen presentation to overcome immunotherapy resistance due to JAK1 loss in melanoma | Kalbasi A | Sci. Transl. Med. | 2020 | 29 |
| 51 | | Use of immunotherapy and surgery for stage IV melanoma | Boland GM | Cancer | 2020 | 2 |
| 52 | | A phase 1/2 trial of an immune-modulatory vaccine against IDO/PD-L1 in combination with nivolumab in metastatic melanoma | Svane IM | Nat. Med. | 2021 | 5 |
| 53 | | A Phase I Study of APX005M and Cabiralizumab with or without Nivolumab in Patients with Melanoma, Kidney Cancer, or Non-Small Cell Lung Cancer Resistant to Anti-PD-1/PD-L1 | Weiss SA | Clin. Cancer Res. | 2021 | 8 |
| 54 | | Adjuvant pembrolizumab versus placebo in resected stage III melanoma (EORTC 1325-MG/KEYNOTE-054): distant metastasis-free survival results from a double-blind, randomised, controlled, phase 3 trial | Eggermont AMM | Lancet Oncol. | 2021 | 37 |
| 55 | | Adjuvant pembrolizumab versus placebo in resected stage III melanoma (EORTC 1325-MG/KEYNOTE-054): health-related quality-of-life results from a double-blind, randomised, controlled, phase 3 trial | Bottomley A | Lancet Oncol. | 2021 | 3 |
| 56 | | Bempegaldesleukin Plus Nivolumab in First-Line Metastatic Melanoma | Diab A | J. Clin. Oncol. | 2021 | 11 |
| 57 | | Biomarker Discovery in Patients with Immunotherapy-Treated Melanoma with Imaging Mass Cytometry | Rimm DL | Clin. Cancer Res. | 2021 | 8 |
| 58 | | Changes in expression of PD-L1 on peripheral T cells in patients with melanoma and lung cancer treated with PD-1 inhibitors | Bowyer SE | Sci Rep | 2021 | 1 |
| 59 | | Chronic Immune-Related Adverse Events Following Adjuvant Anti-PD-1 Therapy for High-risk Resected Melanoma | Johnson DB | JAMA Oncol. | 2021 | 17 |
| 60 | | Cobimetinib plus atezolizumab in BRAF(V600) wild-type melanoma: primary results from the randomized phase III IMspire170 study | Gogas H | Ann. Oncol. | 2021 | 15 |
| 61 | | Combining Nivolumab and Ipilimumab with Infliximab or Certolizumab in Patients with Advanced Melanoma: First Results of a Phase Ib Clinical Trial | Segui B | Clin. Cancer Res. | 2021 | 10 |
| 62 | | CTLA-4 blockade and interferon-a induce proinflammatory transcriptional changes in the tumor immune landscape that correlate with pathologic response in melanoma | Tarhini AA | PLoS One | 2021 | 3 |
| 63 | | Delayed immune-related adverse events with an PD-1-based immunotherapy in melanoma | Long GV | Ann. Oncol. | 2021 | 7 |
| 64 | | Dietary fiber and probiotics influence the gut microbiome and melanoma immunotherapy response | Wargo JA | Science | 2021 | 16 |
| 65 | | Differential effects of PD-1 and CTLA-4 blockade on the melanoma-reactive CD8 T cell response | Kvistborg P | Proc. Natl. Acad. Sci. U. S. A. | 2021 | 1 |
| 66 | | Early Use of High-Dose Glucocorticoid for the Management of irAE Is Associated with Poorer Survival in Patients with Advanced Melanoma Treated with Anti-PD-1 Monotherapy | Boland GM | Clin. Cancer Res. | 2021 | 5 |
| 67 | | Evaluation of TTV replication as a biomarker of immune checkpoint inhibitors efficacy in melanoma patients | Pescarmona R | PLoS One | 2021 | 0 |
| 68 | | Evolution of delayed resistance to immunotherapy in a melanoma responder | Boland GM | Nat. Med. | 2021 | 6 |
| 69 | | FDG PET biomarkers for prediction of survival in metastatic melanoma prior to anti-PD1 immunotherapy | Flaus A | Sci Rep | 2021 | 2 |
| 70 | | Fecal microbiota transplant overcomes resistance to anti-PD-1 therapy in melanoma patients | Zarour HM | Science | 2021 | 142 |
| 71 | | Fecal microbiota transplant promotes response in immunotherapy-refractory melanoma patients | Baruch EN; Markel G | Science | 2021 | 170 |
| 72 | | First-line atezolizumab monotherapy in patients with advanced BRAF(V600) wild-type melanoma | de Azevedo SJ | Pigment Cell Melanoma Res. | 2021 | 2 |
| 73 | | Fructose Promotes Cytoprotection in Melanoma Tumors and Resistance to Immunotherapy | Teague RM | Cancer Immunol. Res. | 2021 | 5 |
| 74 | | G9a Inhibition Enhances Checkpoint Inhibitor Blockade Response in Melanoma | Lee JS | Clin. Cancer Res. | 2021 | 4 |
| 75 | | Germline variants in exonic regions have limited impact on immune checkpoint blockade clinical outcomes in advanced melanoma | Montaudie H | Pigment Cell Melanoma Res. | 2021 | 0 |
| 76 | | Identification of neutralising pembrolizumab anti-drug antibodies in patients with melanoma | Sasson SC | Sci Rep | 2021 | 0 |
| 77 | | Incidence of New Primary Cutaneous Melanoma in Patients With Metastatic Melanoma Treated With Immune Checkpoint Inhibitors A Single-Center Cohort Study | Marchetti MA | JAMA Dermatol. | 2021 | 1 |
| 78 | | Ipilimumab alone or ipilimumab plus anti-PD-1 therapy in patients with metastatic melanoma resistant to anti-PD-(L)1 monotherapy: a multicentre, retrospective, cohort study | Long GV | Lancet Oncol. | 2021 | 18 |
| 79 | | Ipilimumab alone or ipilimumab plus anti-PD-1 therapy in patients with metastatic melanoma resistant to anti-PD-(L)1 monotherapy: a multicentre, retrospective, cohort study | Stratigos AJ | Lancet Oncol. | 2021 | 26 |
| 80 | | Ipilimumab and Radiation in Patients with High-risk Resected or Regionally Advanced Melanoma | Salama AKS | Clin. Cancer Res. | 2021 | 1 |
| 81 | | Long-term outcomes of patients with active melanoma brain metastases treated with combination nivolumab plus ipilimumab (CheckMate 204): final results of an open-label, multicentre, phase 2 study | Tawbi HA | Lancet Oncol. | 2021 | 7 |
| 82 | | Melanoma brain metastasis presentation, treatment, and outcomes in the age of targeted and immunotherapies | Moss NS | Cancer | 2021 | 6 |
| 83 | | Melanoma Cell Intrinsic GABA(A) Receptor Enhancement Potentiates Radiation and Immune Checkpoint Inhibitor Response by Promoting Direct and T Cell-Mediated Antitumor Activity | Sengupta S | Int. J. Radiat. Oncol. Biol. Phys. | 2021 | 5 |
| 84 | | Melanoma dedifferentiation induced by IFN-gamma epigenetic remodeling in response to anti-PD-1 therapy | Ribas A | J. Clin. Invest. | 2021 | 5 |
| 85 | | Mutations in the IFN gamma-JAK-STAT Pathway Causing Resistance to Immune Checkpoint Inhibitors in Melanoma Increase Sensitivity to Oncolytic Virus Treatment | Watson IR | Clin. Cancer Res. | 2021 | 9 |
| 86 | | Neoadjuvant Pembrolizumab and High-Dose IFN alpha-2b in Resectable Regionally Advanced Melanoma | Najjar YG | Clin. Cancer Res. | 2021 | 3 |
| 87 | | Neutral Sphingomyelinase 2 Heightens Anti-Melanoma Immune Responses and Anti-PD-1 Therapy Efficacy | Segui B | Cancer Immunol. Res. | 2021 | 7 |
| 88 | | Nivolumab and Ipilimumab in Metastatic Uveal Melanoma: Results From a Single-Arm Phase II Study | Pelster MS | J. Clin. Oncol. | 2021 | 42 |
| 89 | | Nivolumab Plus Ipilimumab for Treatment-Naive Metastatic Uveal Melanoma: An Open-Label, Multicenter, Phase II Trial by the Spanish Multidisciplinary Melanoma Group (GEM-1402) | Piulats JM | J. Clin. Oncol. | 2021 | 34 |
| 90 | | Pathological response and tumour bed histopathological features correlate with survival following neoadjuvant immunotherapy in stage III melanoma | Scolyer RA | Ann. Oncol. | 2021 | 4 |
| 91 | | Pathway signatures derived from on-treatment tumor specimens predict response to anti-PD1 blockade in metastatic melanoma | Wei Z | Nat. Commun. | 2021 | 0 |
| 92 | | Pembrolizumab Plus Ipilimumab Following Anti-PD-1/L1 Failure in Melanoma | Luke JJ | J. Clin. Oncol. | 2021 | 12 |
| 93 | | Phase I Clinical Trial of Combination Propranolol and Pembrolizumab in Locally Advanced and Metastatic Melanoma: Safety, Tolerability, and Preliminary Evidence of Antitumor Activity | Gandhi S | Clin. Cancer Res. | 2021 | 17 |
| 94 | | Prediction of Immunotherapy Response in Melanoma through Combined Modeling of Neoantigen Burden and Immune-Related Resistance Mechanisms | Chen R | Clin. Cancer Res. | 2021 | 0 |
| 95 | | Standard-Dose Pembrolizumab Plus Alternate-Dose Ipilimumab in Advanced Melanoma: KEYNOTE-029 Cohort 1C, a Phase 2 Randomized Study of Two Dosing Schedules | Long GV | Clin. Cancer Res. | 2021 | 3 |
| 96 | | Survival and biomarker analyses from the OpACIN-neo and OpACIN neoadjuvant immunotherapy trials in stage III melanoma | Blank CU | Nat. Med. | 2021 | 40 |
| 97 | | Targeted Therapy Given after Anti-PD-1 Leads to Prolonged Responses in Mouse Melanoma Models through Sustained Antitumor Immunity | Smalley KSM | Cancer Immunol. Res. | 2021 | 3 |
| 98 | | The impact of BRAF mutation status on clinical outcomes with anti-PD-1 monotherapy versus combination ipilimumab/nivolumab in treatment-naive advanced stage melanoma | Ma VT | Pigment Cell Melanoma Res. | 2021 | 1 |
| 99 | | The PEMDAC phase 2 study of pembrolizumab and entinostat in patients with metastatic uveal melanoma | Ny L | Nat. Commun. | 2021 | 11 |
| 100 | | TMB and Inflammatory Gene Expression Associated with Clinical Outcomes following Immunotherapy in Advanced Melanoma | Hodi FS | Cancer Immunol. Res. | 2021 | 3 |
| 101 | | Transient activation of tumoral DNA damage tolerance pathway coupled with immune checkpoint blockade exerts durable tumor regression in mouse melanoma | Englander EW | Pigment Cell Melanoma Res. | 2021 | 3 |
| 102 | | Type I interferon signaling limits viral vector priming of CD8(+) T cells during initiation of vitiligo and melanoma immunotherapy | Harris JE | Pigment Cell Melanoma Res. | 2021 | 1 |
| 103 | | A PD-1/PD-L1 Proximity Assay as a Theranostic Marker for PD-1 Blockade in Patients with Metastatic Melanoma | Robert C | Clin. Cancer Res. | 2022 | 0 |
| 104 | | Antitumor activity of ipilimumab or BRAF +/- MEK inhibition after pembrolizumab treatment in patients with advanced melanoma: analysis from KEYNOTE-006 | Long GV | Ann. Oncol. | 2022 | 0 |
| 105 | | Circulating Immune Bioenergetic, Metabolic, and Genetic Signatures Predict Melanoma Patients' Response to Anti-PD-1 Immune Checkpoint Blockade | Soto-Pantoja DR | Clin. Cancer Res. | 2022 | 0 |
| 106 | | Cross-cohort gut microbiome associations with immune checkpoint inhibitor response in advanced melanoma | Spector TD | Nat. Med. | 2022 | 1 |
| 107 | | Cutaneous adverse events in 155 patients with metastatic melanoma consecutively treated with anti-CTLA4 and anti-PD1 combination immunotherapy: Incidence, management, and clinical benefit | Patel AB | Cancer | 2022 | 0 |
| 108 | | Early Readout on Overall Survival of Patients With Melanoma Treated With Immunotherapy Using a Novel Imaging Analysis | Dercle L | JAMA Oncol. | 2022 | 2 |
| 109 | | FDG-PET to predict long-term outcome from anti-PD-1 therapy in metastatic melanoma | Menzies AM | Ann. Oncol. | 2022 | 1 |
| 110 | | Frailty and checkpoint inhibitor toxicity in older patients with melanoma | Bruijnen CP | Cancer | 2022 | 0 |
| 111 | | Intestinal microbiota signatures of clinical response and immune-related adverse events in melanoma patients treated with anti-PD-1 | Zarour HM | Nat. Med. | 2022 | 0 |
| 112 | | Long-Term Outcomes With Nivolumab Plus Ipilimumab or Nivolumab Alone Versus Ipilimumab in Patients With Advanced Melanoma | Wolchok JD | J. Clin. Oncol. | 2022 | 9 |
| 113 | | Multiomic profiling of checkpoint inhibitor-treated melanoma: Identifying predictors of response and resistance, and markers of biological discordance | Long GV | Cancer Cell | 2022 | 1 |
| 114 | | Neoantigen-reactive CD8(+) T cells affect clinical outcome of adoptive cell therapy with tumor-infiltrating lymphocytes in melanoma | Hadrup SR | J. Clin. Invest. | 2022 | 0 |
| 115 | | Phase II study of ceralasertib (AZD6738) in combination with durvalumab in patients with advanced/metastatic melanoma who have failed prior anti-PD-1 therapy | Lee J | Ann. Oncol. | 2022 | 5 |
| 116 | | Relatlimab and Nivolumab versus Nivolumab in Untreated Advanced Melanoma | Tawbi HA | N. Engl. J. Med. | 2022 | 14 |
| 117 | | T cell characteristics associated with toxicity to immune checkpoint blockade in patients with melanoma | Chaudhuri AA | Nat. Med. | 2022 | 1 |
| 118 | | Vitiligo-specific soluble biomarkers as early indicators of response to immune checkpoint inhibitors in metastatic melanoma patients | Carbone ML | Sci Rep | 2022 | 0 |
